# Global prevalence of multimorbidity during pregnancy: a systematic review and meta-analysis

**DOI:** 10.1186/s12978-026-02424-8

**Published:** 2026-07-27

**Authors:** Lena Elise Wessing, Shrouq Daraiseh, Yasmin Semmane, Adamantia Styliani Chassioti, Adhithi Sreenivasan, Veronika Tirado, Emily White Johansson, Renee Gardner, Sibylle Herzig van Wees, Claudia Hanson, Sunjuri Sun

**Affiliations:** 1 Department of Global Public Health, Karolinska Institutet, Stockholm, Sweden; 2 Department of Public Health Sciences, Stockholm University, Stockholm, Sweden; 3 Swedish Centre for Impacts of Climate Extremes (climes), Karolinska Institutet, Stockholm, Sweden; 4 Department of Women’s and Children’s Health, Global Health and Migration Unit, Uppsala University, Uppsala, Sweden; 5 London School of Hygiene and Tropical Medicine, London, UK; 6 Department of Global Public Health, Widerströmska Huset, Karolinska Institutet, Tomtebodavägen 18, Solna, 171 65 Sweden

## Abstract

**Background:**

Multimorbidity, defined as having two or more coexisting physical or mental health conditions, increases the risk of complications and need of specialist care during pregnancy. In women of reproductive age, pregnancy represents a critical period during which multimorbidity may manifest or be exacerbated. Multimorbidity during pregnancy has been associated with a wide range of adverse maternal and neonatal outcomes. This systematic review aims to estimate the global and regional prevalences of multimorbidity during pregnancy, as well as to understand how multimorbidity is defined across studies.

**Methods:**

We systematically searched four databases (Medline, Embase, CINAHL and Web of Science) for observational studies published from January 1, 2015, to September 26, 2025, without language or geographic restrictions. The protocol was registered in the PROSPERO database (CRD420251153876).

We included peer-reviewed cross-sectional and cohort studies including ≥ 200 pregnant women, reporting the prevalence of multimorbidity. Data screening, extraction and quality assessment were conducted independently by two reviewers following PRISMA 2020 and Joanna Briggs Institute guidelines. Pooled prevalence was estimated via random-effects meta-analysis, and explored through predefined subgroup analyses.

**Results:**

The literature search retrieved 6,243 studies, and after title/abstract and full-text screening, 18 observational studies across eight countries were included in the review. The pooled prevalence of multimorbidity during pregnancy was 7.18% (95% CI: 3.31-14.89, I2 = 100.0%), with regional differences: 1.74% (95% CI: 0.46-6.37, I2 = 99.98%) in Asia, 9.07% (95% CI: 3.01-24.30, I2 = 100.0%) in North America, 11.17% (95% CI: 1.22-56.18, I2 = 99.94%) in Oceania and 12.64% (95% CI: 3.41-37.24, I2 = 99.99%) in Europe. However, the wide confidence intervals for Europe and Oceania warrant cautious interpretation of regional comparisons.

**Conclusion:**

Multimorbidity during pregnancy is a significant and under-researched global health concern. The pooled prevalence of 7.18% reflects a synthesis of highly heterogeneous studies and should not be interpreted as a definitive estimate of the global burden. More standardized research, particularly in low- and middle-income countries, is needed to generate more representative prevalence estimates.

**Supplementary Information:**

The online version contains supplementary material available at 10.1186/s12978-026-02424-8.

## Background

Multimorbidity, defined as the co-existence of at least two chronic physical or mental health conditions, has emerged as a significant global public health concern. Historically, health systems, research and clinical guidelines have largely been organized around single-disease frameworks [1]. However, the growing burden of chronic conditions worldwide has led to recognition of multimorbidity as a distinct health challenge. Individuals with multimorbidity often experience poorer health outcomes, increased healthcare utilization and fragmented care, underscoring the need for integrated care models [1].

Most prevalence estimates of multimorbidity come from studies in older populations. A systematic review by Chowdhury et al. reported a global pooled prevalence of multimorbidity of 39.4% among non-pregnant adult women aged 30 or above, with regional variation from 33.3% in Africa to 50.1% in South America [2]. However, these estimates are based on varying operational definitions of multimorbidity, including differences in the number and types of conditions considered, and should therefore be interpreted with caution.

In contrast, global estimates of multimorbidity among pregnant women remain limited, and are further complicated by substantial heterogeneity in how the concept is defined and operationalized in this population. Studies differ in their inclusion of chronic and acute conditions, pregnancy-specific complications, and mental health disorders, all of which may influence prevalence estimates and limit comparability across settings. Beyond the variation in multimorbidity definitions, reported prevalence estimates are likely shaped by structural differences between settings: while high-income countries (HICs) generally benefit from well-established antenatal screening programs, many low- and middle income countries (LMICs) face fragmented health systems, suboptimal adherence to antenatal care standards and underreporting of non-communicable diseases (NCDs) during pregnancy, potentially leading to underestimation of the true burden of multimorbidity [3, 4]. Despite these challenges, pregnancy represents a critical period during which multimorbidity may manifest or be exacerbated, potentially leading to adverse maternal and neonatal outcomes [5], and a need for more intensive monitoring, coordinated care across specialties, and individualized management plans [6, 7].

Multimorbidity often involves both physical and mental health conditions [8]. NCDs are major contributors to maternal mortality and morbidity [5, 9]. In HICs, common conditions among pregnant women include mental health disorders, obesity and respiratory disorders [8, 10]. In LMICs, both communicable and non-communicable diseases contribute to the burden of multimorbidity, with anemia, HIV and depression reported as predominant maternal morbidities in these settings [11]. Notably, pregnancy-induced hypertension and diabetes occur across all settings [12]. A systematic review by Sreenivasan et al. found higher prevalences of mental-health related multimorbidity during pregnancy in lower-middle income countries (10.29%) and low-income countries (LICs) (9.44%), compared to HICs (1.06%) [13].

Multimorbidity during pregnancy has been associated with a wide range of adverse maternal and neonatal outcomes, including maternal mortality, severe morbidity (such as preeclampsia), preterm birth, low birth weight and cesarean delivery [9, 14, 15]. Beyond the perinatal period, multimorbidity increases the risk of persistent NCDs in women and adverse long-term outcomes in offspring [5]. For example, gestational diabetes has been linked to the development of type 2 diabetes mellitus in mothers later in life [16]. These complications lead to higher healthcare utilization and expenditures [17], both during and after pregnancy [5]. These adverse effects are likely most pronounced in LMICs, due to the triple burden of high fertility rates, high maternal and neonatal mortality and poor maternal health [18].

Although multimorbidity during pregnancy has received less attention in research than multimorbidity in older adult populations, it is becoming increasingly relevant in the context of changing maternal demographics and evolving disease patterns [5]. As women today are generally older at first childbirth due to changing societal roles and increased life expectancy, they enter pregnancy with a higher burden of pre-existing conditions [18]. Concurrently, conditions once associated with older age are now emerging earlier in life, including during the reproductive years [5]. While primary studies have reported prevalence estimates in specific populations [6, 19–21], no systematic review has comprehensively synthesized global and regional estimates or examined how multimorbidity during pregnancy is defined across studies. Therefore, this systematic review aims to estimate the global and regional prevalences of multimorbidity during pregnancy, as well as to understand how multimorbidity is defined across studies.

## Methods

### Search strategy and selection criteria

We searched four online databases on 26 September 2025: Medline (Ovid), Embase, CINAHL and Web of Science. Eligibility criteria for inclusion were studies that (1) had pregnant women as population, (2) provided an estimated prevalence of multimorbidity with at least two coexisting conditions (including chronic pre-existing diseases as well as pregnancy-related conditions, such as gestational diabetes) during pregnancy as an outcome, (3) provided a definition of multimorbidity used in the study, (4) were original peer-reviewed cross-sectional or cohort studies, (5) included at least 200 pregnant participants, and (6) were published after January 1, 2015. Studies with at least 200 pregnant participants were included to ensure sufficient precision of prevalence estimates, consistent with established principles that larger samples yield narrower confidence intervals and more reliable proportions in prevalence research [22]. The 10-year window was selected to aim for diagnostic consistency across included studies, as 2015 marked the mandatory transition from ICD-9-CM to ICD-10-CM diagnostic coding, which substantially altered how maternal morbidities are identified and recorded in administrative data [23]. Furthermore, the epidemiology of pregnancy has shifted substantially over the last few decades, with increasing maternal age at first birth and rising burden of chronic diseases [24, 25]. Thus, this restriction ensures that the findings of this systematic is relevant to contemporary maternal care. Finally, multimorbidity during pregnancy has only recently gained traction in the scientific literature, including conceptualization and operationalization of definitions and frameworks for multimorbidity in pregnancy [15, 21, 26, 27]. Thus, the rationale for restricting studies published from 2015 was to improve comparability across studies due to changes in diagnostic coding practices, the evolving epidemiology of pregnancy and recent formalization of the concept of multimorbidity during pregnancy [13]. Exclusion criteria were studies (1) without pregnant women, (2) not reporting multimorbidity prevalences, (3) only focusing on comorbidity, single diseases or an index condition (condition that serves as a reference point), (4) without supporting original data, and (5) with any other study design than a cross-sectional or cohort design. Studies were included regardless of language, geographical location or setting, to accurately estimate the global prevalence.

The systematic review and meta-analysis have been reported conforming to the Preferred Reporting Items for Systematic Review and Meta-Analysis (PRISMA) 2020 reporting guidelines [28] and Joanna Briggs Institute (JBI) framework for systematic reviews [29] (Supplement 2), with the protocol registered in PROSPERO (CRD420251153876). Supplement 1 describes the search strategy.

### Data analysis

All records were stored in Mendeley, then transferred to Covidence for de-duplication, screening, data extraction and risk of bias assessment. Duplicates were manually reviewed by one reviewer (LW). Two independent reviewers (LW, SD, YS, SS) screened titles and abstracts in Covidence against the eligibility criteria. This was repeated for full-text screening (LW, SD, YS, SS, AC). If full texts were not publicly available, we requested them through the Karolinska Institutet Library services. Data extraction and quality assessment was conducted using Covidence software by two independent reviewers (LW, SD, YS, SS, AC). Conflicts during screening and data extraction were resolved through consensus or a third reviewer. The extraction table for the full-text data extraction can be found in Supplement 3.

The JBI Critical Appraisal Tools Checklist for Prevalence Studies [30] has been used to assess quality of the included studies. The tool consisted of nine questions, categorized into three domains: “participant selection”, “methods” and “measurement of outcome” (Supplement 4). Each question could be answered with “Yes” (2 points), “No” (0 points), “Unclear” (1 point) and “Not Applicable” (1 point). Although the JBI checklist was not originally designed as a quantitative scoring instrument, JBI does not prescribe fixed cut-off scores, and the adaptation of checklist items into researcher-defined summed quality scores is an established practice in the systematic review literature [31, 32]. The quality was calculated as high quality (17–18 points), moderate quality (14–16 points) or low quality ($\:\le\:$13 points).

We performed statistical analysis using *meta* packages in the R statistical software (version 4.5.0). The prevalence of multimorbidity during pregnancy per study has been estimated as a proportion with the number of pregnant women experiencing multimorbidity as the numerator, and the total sample size as the denominator. We calculated the global pooled prevalence of multimorbidity during pregnancy through meta-analysis using a random-effects generalized linear mixed model with a logit transformation, and visually represented both the proportion and 95% CI with forest plots. To assess heterogeneity across the included studies, we ascertained the *I*^*2*^ and τ² statistic, which indicates the proportion of total variation across studies due to heterogeneity rather than chance. Publication bias was assessed using funnel plots and Egger’s regression test. Funnel plot asymmetry was assessed visually, and Egger’s test was used to statistically detect small-study effects. We have performed subgroup analyses by determining the pooled prevalence for the following subgroups: geographical region, study year, study design, sample size and number of conditions in multimorbidity definitions. Additionally, we performed sensitivity analyses to assess the robustness of our findings.

## Results

### Selection of studies

**Fig. 1:**
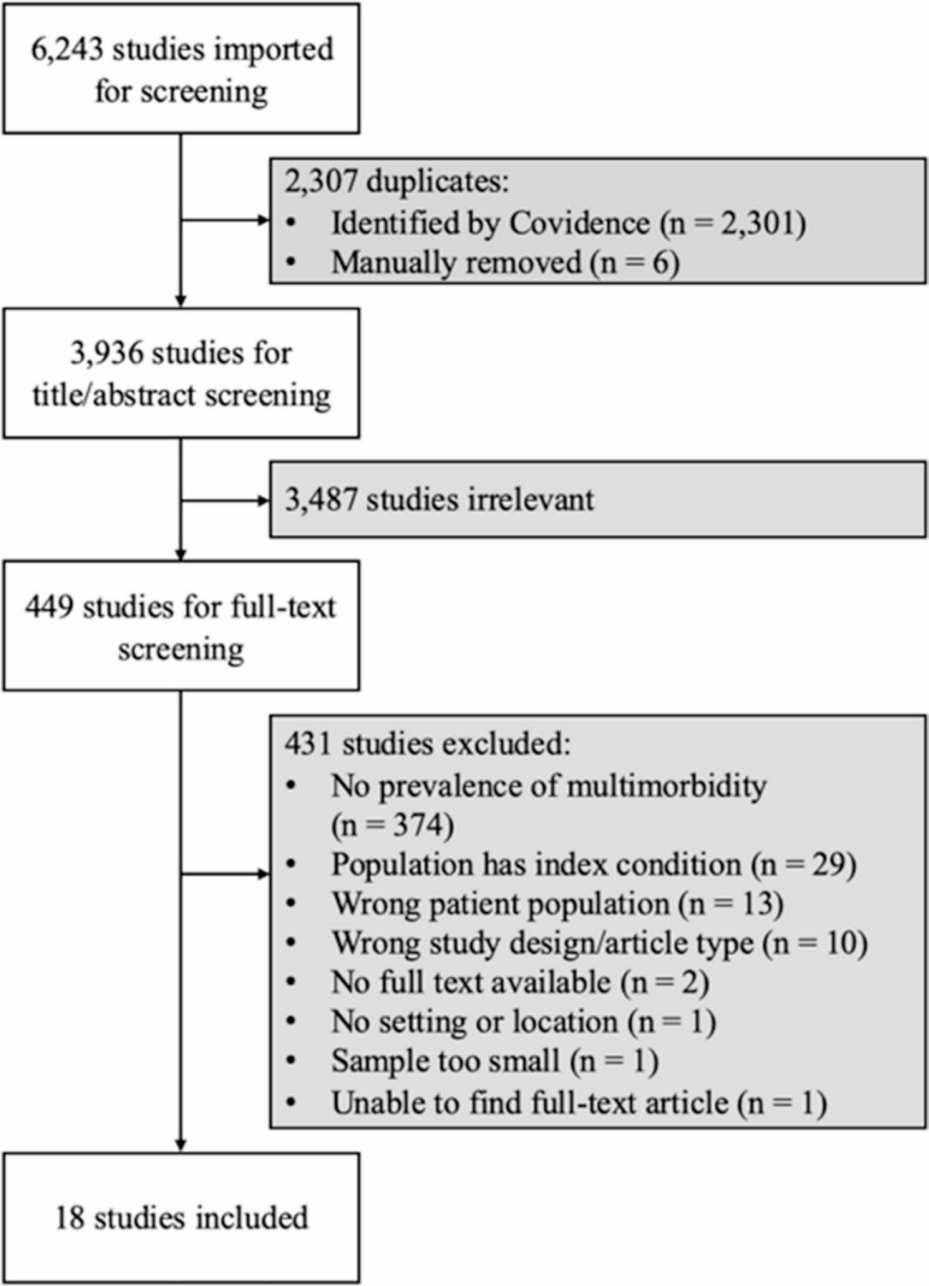
Flowchart of literature search and article selection

A flowchart summarizing the literature search and article selection process is presented in Fig. 1. The initial searches acquired 6,243 studies from the four databases, which included 2,307 duplicate studies. After title- and abstract screening, 449 studies were selected for full-text screening and 18 studies were included in the systematic review and meta-analysis, based on the eligibility criteria.

### Characteristics of the studies

**Fig. 2:**
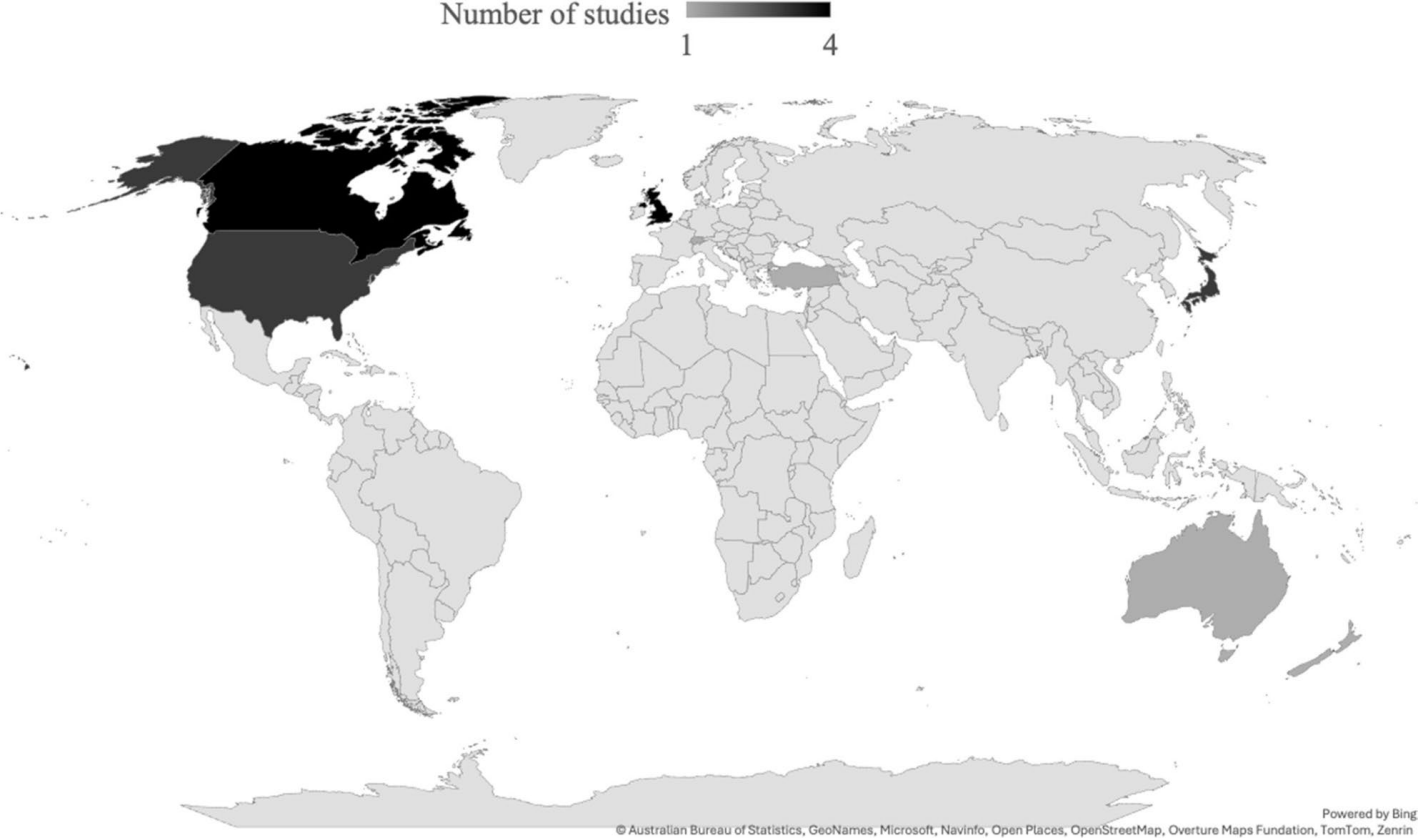
Included studies per country

The key characteristics of the included studies are shown in Table 1 and Supplement 5. In total, 18 observational studies were analyzed, spanning across 8 countries and 4 continents (Fig. 2) and covering data from 13,900,016 pregnant women. Geographically, most studies were carried out in North America (*n* = 7), followed by Europe (*n* = 5), Asia (*n* = 4) and Oceania (*n* = 2). Regarding economic classification [33], 17 studies were conducted in HICs, one in an upper-middle-income country (UMIC), and no studies were found in lower-middle-income countries and LICs. In total, there were 16 cohort and 2 cross-sectional studies. Data collection ranged from 2000 to 2021, and studies had patient populations ranging from 693 to 7,696,836 pregnant women.

**Table 1:** Characteristics of included studies

Author and year (ref)	Country	World Bank income group	Study design	Study start – end year	Source of data	Method of recruitment	Sample size (*N*)	Number of conditions in definition list	Point in time of multimorbidity ascertainment	Prevalence of multimorbidity (*n* (%))
Akagi 2024 [34]	Japan	High	Cohort	2011–2014	The Japan Environment and Children’s Study	Routinely collected data	82,877	23	Those that were medically treated at the time of pregnancy, point in time not specified	3,001 (3.6%)
Akaishi 2023 [35]	Japan	High	Cohort	2016–2021	Nationwide Diagnosis Procedure Combination (DPC) database	Routinely collected data	804,617	9	Before admission for delivery and at readmission for postpartum depression/suicide attempt	1,502 (0.19%)
Aubry 2019 [36]	Switzerland	High	Cohort	2005–2016	Arbeitsgemeinschaft Schweizerischer Frauenkliniken (ASF) Database Switzerland	Routinely collected data	324,664	6	Not specified, pre-pregnancy and during pregnancy	2,170 (0.7%)
Aumais 2025 [37]	Canada	High	Cohort	2018 − 2014	Perinatal Multisite Databank (PMD)	Clinic patients	693	12	While pregnant or within 6 months after giving birth to a live baby.	325 (46.9%)
Azcoaga-Lorenzo 2023 [15]	Scotland (UK)	High	Cohort	2014–2018	EHR from Scottish Morbidity Records	Clinic patients	27,771	79	At the estimated time of conception	4,666 (16.8%)
Belsti 2025 [38]	Australia	High	Cross sectional	2016–2021	Monash Health maternity hospitals in Melbourne	Routinely collected data	48,502	25	During pregnancy	19,469 (40.1%)
Brown 2024 [10]	Canada	High	Cohort	2007–2020	Institute for Clinical Evaluative Sciences	Routinely collected data	2,014,508	22	In 5-year period before the estimated date of conception for index pregnancy	324,735 (16.1%)
Brown 2025 [17]	Canada	High	Cohort	2012–2021	Linked “ICES” and “Better Outcomes Registry and Network (BORN)” data Ontario	Routinely collected data	1,373,193	23	In the two years before the estimated date of conception	121,753 (8.9%)
Chhabria 2024 [39]	USA	High	Cohort	2014–2019	Clinformatics Data Mart Database (CDM)	Routinely collected data	372,895	9	9 months prior to delivery and up to 4 months after delivery	16,630 (4.47%)
Dikmen-Yildiz 2017 [40]	Turkey	Upper-middle	Cohort	2014–2015	Turkish Statistical Institute	Clinic patients	950	3	At intake (gestational age 26–35 weeks), 4–6 weeks postpartum and 6 months postpartum	20 (2.1%)
Hohmann-Marriott 2019 [41]	New Zealand	High	Cohort	2008–2009	Growing Up in New Zealand (GUiNZ) cohort	Clinic patients	6,822	6	Before this pregnancy and during this pregnancy	157 (2.3%)
Kent 2025 [8]	Northern Ireland (UK)	High	Cohort	2012–2020	Northern Ireland Regional Maternity Service Database (NIMATS) + Health and Care Number (HCN) + Family Practitioner Services Enhanced Prescribing Database (EPD)	Routinely collected data	137,750	79	In period 12 months prior to conception	30,535 (22.17%)
Lau 2025 [42]	USA	High	Cohort	2001–2019	Predictive Analysis with Deep Learning Models for Maternal Endpoints (PADME) cohort	Routinely collected data	56,833	14	From one year preceding the start of pregnancy episode to one year postpartum	191 (0.3%)
Lee 2022 [21]	UK	High	Cross sectional	2018	Routine healthcare datasets from primary care (CPRD)	Routinely collected data	37,641	79	Prior to the index pregnancy	16,637 (44.2%)
Nakanishi 2023 [43]	Japan	High	Cohort	2011–2014	Japan Environment and Children’s Study (JECS)	Clinic patients	86,885	23	Conditions treated from pregnancy diagnosis to 12 weeks of gestation	5,462 (6.3%)
Stanhope 2022 [44]	USA	High	Cohort	2014–2021	Grady Obstetric and Gynecologic Outcomes (GOGO) database	Routinely collected data	14,225 births to 12,409 people	11	Comorbidities diagnosed up to 1 year before the index birth and readmissions occurring up to 90 days after the index birth	1,436 (10.1%)
Subramanian 2023 [45]	UK	High	Cohort	2000–2019	Pregnancy Register within Clinical Practice Research Datalink (CPRD) GOLD	Routinely collected data	812,354	79	At the point of conception, or treated over the past 12 months in the case of eczema	139,471 (17.2%)
Thompson 2022 [46]	USA	High	Cohort	2001–2012	Centers for Medicare and Medicaid Services Analytic eXtract (MAX) Database	Routinely collected data	7,696,836	27	2 year look-back period	1,940,587 (25.2%)

### Global and regional prevalences of multimorbidity during pregnancy

**Fig. 3:**
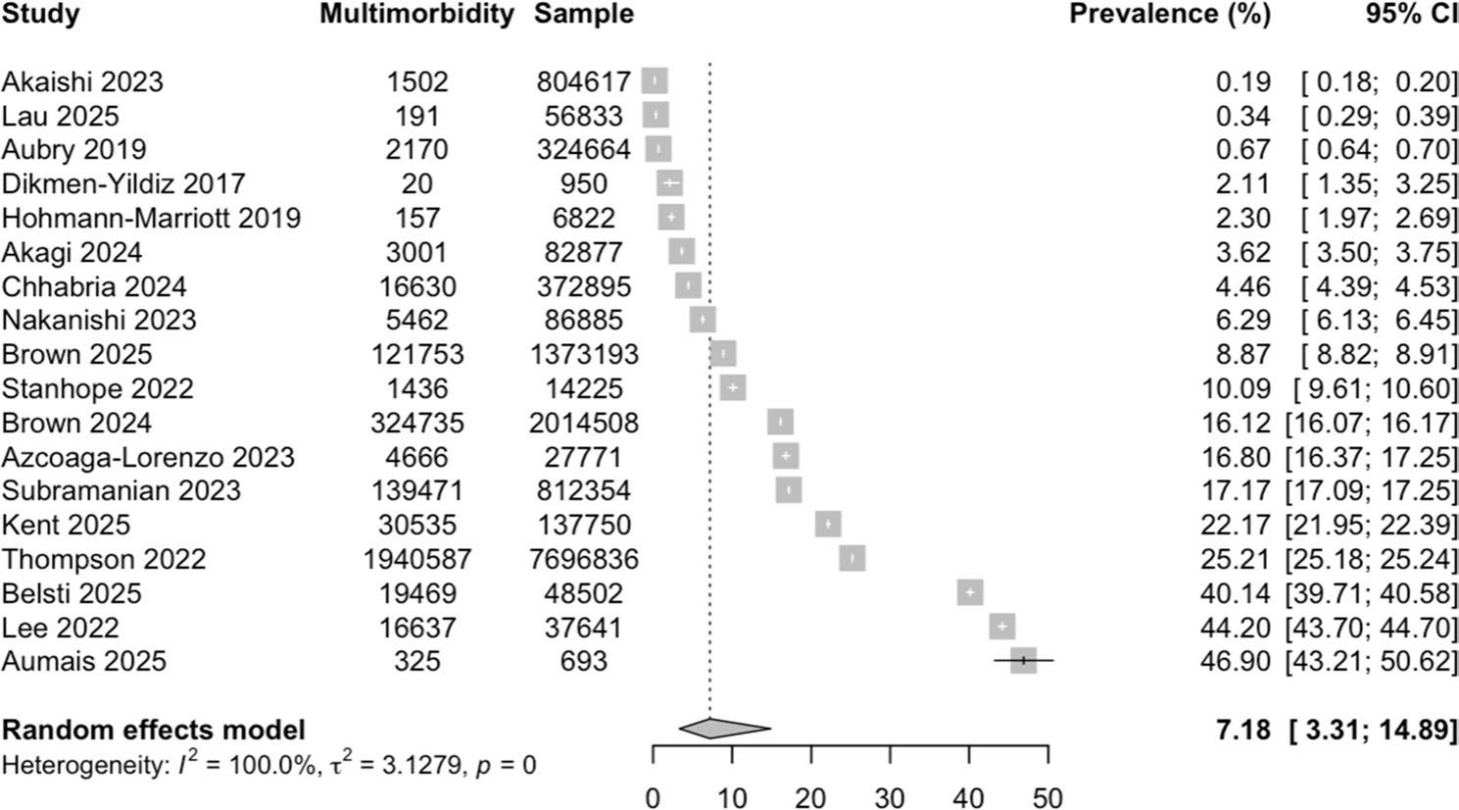
Forest plot of the overall prevalence of multimorbidity among pregnant women

The random-effects pooled prevalence of multimorbidity during pregnancy, estimated from the 18 included studies, was 7.18% (95% CI: 3.31–14.89, τ² = 3.128, I^2^ = 100.0%). The prevalence of multimorbidity among pregnant women in the included studies ranged from 0.19% to 46.90%. Prevalences and estimated confidence intervals are shown in a forest plot (Fig. 3). Given the extreme heterogeneity observed (I^2^ = 100.0%), the pooled estimates should be interpreted with considerable caution, as the included studies fundamentally differ in study population, multimorbidity definition, and methodology. The pooled estimates should be viewed as descriptive summary measures of reported prevalence estimates across diverse settings rather than a definitive epidemiological prevalence estimate.

At the regional level, the pooled prevalence of multimorbidity was highest in Europe with 12.64% (95% CI: 3.41–37.24, τ² = 2.600, I^2^ = 99.99%), followed by Oceania with 11.17% (95% CI: 1.22–56.18, τ² = 2.805, I^2^ = 99.94%), North America with 9.07% (95% CI: 3.01–24.30, τ² = 2.671, I^2^ = 100.00%) and Asia with 1.74% (95% CI: 0.46–6.37, τ² = 1.867, I^2^ = 99.98%) (Table 2 and Supplement 7 A). The extremely wide confidence intervals, particularly for Europe and Oceania, and the substantial between-study variance across all regions may reflect variation in methodologies or sparse evidence rather than true geographic differences, therefore regional estimates should similarly be interpreted with caution.

**Table 2:** Subgroup analysis

Subgroup		N of studies (*n* = 18)	Pooled prevalence of multimorbidity	95% CI	I^2^ (%)
*Geographical location*	North America South America Africa	7 0 0	9.07% - -	3.01–24.30 - -	100.00% - -
	Europe	5	12.64%	3.41–37.24	99.99%
	Asia	4	1.74%	0.46–6.37	99.98%
	Oceania	2	11.17%	1.22–56.18	99.94%
*Study start year*	Before 2010	6	4.21%	1.03–15.63	100.00%
	2010–2014	8	7.42%	4.40–12.26	99.98%
	2015 or later	4	14.68%	1.31–69.02	99.99%
*Study design*	Cross-sectional	2	42.15%	39.37–44.99	99.30%
	Cohort	16	5.52%	2.50–11.78	100.00%
*Sample size*	200-9,999	3	7.09%	1.05–35.36	99.82%
	10,000–99,999	7	9.12%	2.71–26.59	99.99%
	100,000–999,999	5	3.12%	0.58–15.13	100.00%
	$\:\ge\:$1,000,000	3	15.59%	9.40–24.76	100.00%
*Number of conditions that could contribute to the multimorbidity definition*	3–9 conditions	5	1.22%	0.45–3.23	99.98%
	10–19 conditions	3	6.48%	0.51–48.31	99.93%
	$\:\ge\:$ 20 conditions	10	16.64%	10.20–25.97	100.00%
*Types of conditions*	*Chronic mental and physical*	12	11.32%	6.84–18.15	100.00%
	*Chronic mental*	3	3.16%	0.18–36.74	100.00%
	*Chronic and pregnancy-specific physical*	3	2.42%	0.17–26.08	100.00%

### Subgroup analysis

Subgroup analysis by geographical region, study year, study design, sample size and number of conditions in multimorbidity definitions is shown in Table 2. Given the insufficient representation of different income levels, subgroup analysis by World Bank income group was not feasible.

#### Study start year

Studies starting before 2010 reported the lowest pooled prevalence at 4.21% (95% CI: 1.03–15.63, τ² = 3.201, I^2^ = 100.00%) increasing to 7.42% (95% CI: 4.40–12.26, τ² = 0.637, I^2^ = 100.00%) for 2014–2014, and further rising to 14.68% (95% CI: 1.31–69.02, τ² = 6.826, I^2^ = 100.00%) for studies from 2015 onward (Supplement 7B). Prevalence estimates tended to increase with more recent study start years, although estimates for the most recent studies were highly variable, as reflected by the large τ² and wide confidence interval.

#### Study design

Cross-sectional studies showed a substantially higher pooled prevalence (42.15%, 95% CI: 39.37–44.99, τ² = 0.007, I^2^ = 99.30%) compared to cohort studies (5.52%, 95% CI: 2.50–11.78, τ² = 2.811, I^2^ = 100.00%) (Supplement 7C). However, the subgroup estimate for cross-sectional studies is based on only two studies and should therefore be interpreted with caution due to limited precision and potential instability of the pooled estimate.

#### Sample size

No clear pattern was observed between sample size and prevalence estimates. Smaller studies with 200-9,999 and 10,000–99,999 reported pooled prevalences of 7.09% (95% CI: 1.05–35.36, τ² = 3.009, I^2^ = 99.80%) and 9.12% (2.71–26.59, τ² = 3.000, I^2^ = 100.00%), respectively. Studies with 100,000–999,999 pregnant participants reported the lowest prevalence at 3.12% (95% CI: 0.58–15.13, τ² = 3.782, I^2^ = 100.00%), while studies with $\:\ge\:\:$1,000,000 pregnant participants reported the highest prevalence at 15.59% (95% CI: 9.40–24.76, τ² = 0.258, I^2^ = 100.00%) (Supplement 7D).

#### Number of conditions that could contribute to the definition of multimorbidity

The number of conditions included in the multimorbidity definition substantially influenced prevalence estimates (Supplement 7E). Studies examining 3–9 conditions reported the lowest prevalence at 1.22% (95% CI: 0.45–3.23, τ² = 1.285, I^2^ = 100.00%), while those considering $\:\ge\:$ 20 conditions reported a nearly 10-fold increase in prevalence at 16.64% (95% CI: 10.20–25.97, τ² = 0.827, I^2^ = 100.00%). Studies examining 10–19 conditions reported intermediate estimates at 6.48% (95% CI: 0.51–48.31, τ² = 5.281, I^2^ = 99.90%).

#### Types of conditions

Prevalence estimates also varied according to the types of conditions included in the multimorbidity definition (Supplement 7F). Studies including both chronic mental and physical conditions reported the highest pooled prevalence at 11.32% (95% CI: 6.84–18.15, τ² = 0.948), while studies including chronic mental conditions alone or a combination of chronic and pregnancy-specific physical conditions reported pooled prevalences of 3.16% (95% CI: 0.18–36.74, τ² = 6.428) and 2.42% (95% CI: 0.17–26.08, τ² = 5.521), respectively. However, differences between subgroups were not statistically significant (*p* = 0.34), and substantial heterogeneity remained within each subgroup (I^2^ = 100%).

High heterogeneity was observed across all subgroups (I^2^ > 99.90% for all analyses), indicating substantial variation in study characteristics, populations and methodologies that was not explained by the examined subgroup variables alone. These trend estimates should therefore be interpreted with caution.

### Publication bias

The Egger’s test suggested evidence of small-study effects (*p* = 0.032) among the 18 included studies evaluating the prevalence of multimorbidity among pregnant women. There is evidence of funnel plot asymmetry (Supplement 8), which may suggest publication bias, but could also be explained by the high heterogeneity with all I^2^ above 99.90%. We consequently applied a trim-and-fill analysis to adjust for the possible publication bias. Trim-and-fill analysis imputed seven potentially missing studies with higher prevalences of multimorbidity among pregnant women. Incorporating these studies increased the pooled prevalence to 21.22% (95% CI: 8.95–42.49, I^2^ = 100.00%, τ² = 6.616), markedly higher than the original estimate of 7.18% (95% CI: 3.31–14.89, I^2^ = 100.00%, τ² = 3.128). However, given the profound methodological heterogeneity across studies, the observed asymmetry and trim-and-fill results should be interpreted cautiously, as they may reflect genuine between-study differences rather than selective publication. Furthermore, the direction of potential asymmetry, suggesting missing high-prevalence studies, is inconsistent with traditional publication bias patterns and more likely reflects population heterogeneity. Because high heterogeneity can generate funnel plot asymmetry unrelated to publication bias and the relatively small number of studies (*n* = 18) makes the Egger’s test sensitive to outliers, publication bias cannot be assumed.

### Sensitivity analysis

Several sensitivity analyses were conducted to assess the robustness of the pooled prevalence estimates, with forest plots reported in Supplement 9. First, we excluded studies with sample sizes below 10,000 participants (*n* = 3) to evaluate potential small-study effects (Supplement 9A). This led to a pooled prevalence of 7.20% (95% CI: 3.05–16.06, τ² = 3.151), marginally higher than the pooled prevalence in the original meta-analysis (7.18%), suggesting minimal influence of smaller studies on the overall estimate. When restricting the analysis to cohort studies only (*n* = 16), the pooled prevalence decreased to 5.52% (95% CI: 2.51–11.73, τ² = 2.811) (Supplement 9B). Conversely, excluding studies that defined multimorbidity using fewer than 10 conditions (*n* = 5) resulted in a substantially higher pooled prevalence of 13.53% (95% CI: 6.69–25.45, τ² = 2.049) (Supplement 9C). Similarly, only including studies that looked at both chronic mental and physical conditions (*n* = 12) resulted in a higher pooled prevalence of 11.32% (95% CI: 6.84–18.15, τ² = 0.948) (Supplement 9D). Moreover, when excluding the one study from a UMIC and only analyzing HICs, the pooled prevalence increased to 7.70% (95% CI: 3.43–16.39, τ² = 0.769) (Supplement 9E). Although all sensitivity analyses maintained considerable heterogeneity (I^2^ = 100.00%), the between-study variance (τ²) decreased when analyses were restricted to more homogeneous study characteristics, particularly studies including both chronic mental and physical conditions, and studies conducted in HICs.

Additionally, we performed an outlier analysis by excluding studies with prevalence estimates beyond one standard deviation from the pooled estimate (< 1.30% or > 31.20%), removing six studies. This restriction increased the pooled prevalence to 8.67% (95% CI: 5.35–13.76, I^2^ = 100.00%, τ² = 3.21) (Supplement 9F).

## Discussion

In this systematic review and meta-analysis, we analyzed data from 18 studies covering nearly 14 million pregnant women across 8 countries and 4 continents to estimate the global prevalence of multimorbidity during pregnancy. The pooled prevalence of multimorbidity during pregnancy in this study was 7.18% (95% CI: 3.31–14.89, I^2^ = 100.0%). Given the extreme between-study heterogeneity, this pooled estimate should be interpreted as a descriptive summary of the included studies rather than a definitive estimate of the global pooled prevalence of multimorbidity during pregnancy. The wide range of prevalence estimates across individual studies (0.19% to 46.90%) reflects substantial geographical and methodological variability, thus limiting the interpretability and comparability of pooled estimates.

As anticipated in systematic reviews and meta-analyses of prevalence [47], high heterogeneity was observed across all analyses (I^2^ > 99.90%). This was also reflected in substantial between-study variance (τ² values ranging from approximately 0.8 to 6.8 across analyses), indicating large absolute differences in prevalence estimates across studies. The wide variation in prevalence estimates (0.19%−46.9%) appears largely driven by methodological differences rather than true population variation, particularly heterogeneity in condition lists (ranging from 3 to 79 conditions), data sources, recruitment strategies, and ascertainment periods. In particular, inconsistent temporal ascertainment windows, ranging from chronic pre-conception and pregnancy-only to inclusion of postpartum conditions, introduce important conceptual differences in what is defined as pregnancy-related multimorbidity, potentially affecting comparability across studies and pooling decisions. To address the high heterogeneity, we applied a random-effects model and conducted subgroup and sensitivity analyses, although residual heterogeneity remained. This heterogeneity persisted across all subgroup analyses, suggesting that the variation stems from fundamental differences in study design, population characteristics and healthcare contexts. Accordingly, we conducted subgroup analyses and sensitivity analyses to investigate potential drivers of heterogeneity. Substantial heterogeneity in prevalence estimates was explained primarily by variation in multimorbidity definitions, which represents an important finding. Studies using narrow definitions (3–9 conditions) reported a pooled prevalence of 1.22%, while those using comprehensive definitions (≥ 20 conditions) reported 16.64%: an almost 10-fold difference, driven entirely by methodological choices rather than true epidemiological variation. Studies that included both chronic mental and physical health conditions similarly reported a higher pooled prevalence at 11.32%, while those only including chronic mental health conditions or a combination of chronic and pregnancy-specific physical health conditions reported lower prevalences at 3.16% and 2.42, respectively. Similarly, the two cross-sectional studies showed a substantially higher pooled prevalence (42.15%), compared to the 16 cohort studies (5.52%). Despite high heterogeneity, the subgroup analyses underline the critical need for standardized multimorbidity definitions in epidemiological research.

Regional variations in multimorbidity prevalence among pregnant women were considerable, with the highest estimates observed in Europe (12.64%) and Oceania (11.7%), followed by North America (9.07%) and Asia (1.74%). These differences could reflect variations in healthcare systems, diagnostic practices, population demographics, health-seeking behaviour and study methodologies rather than true epidemiological differences alone [2]. However, the extremely wide confidence intervals, especially for Europe and Oceania, underscore the uncertainty in these regional estimates and the need for cautious interpretation. Additionally, the continent-level estimates are driven by a small number of contributing studies that are geographically not representative of the regions as a whole. In particular, European and Asian estimates are largely based on studies from the UK and Japan, respectively, limiting the validity of broader continental generalizations. Accordingly, these findings are more accurately interpreted as reflecting country-specific contexts rather than true regional differences. Temporal trends also showed increasing prevalence over time, rising from 4.21% before 2010 to 14.68% after 2015. A similar trend has also been shown in an United States serial cross-sectional analysis by Admon et al., who reported an increase in prevalence of multimorbidity among pregnant women over time, ranging from 0.47% in 2005 to 0.81% in 2014 [48]. However, these findings should be interpreted with caution, as observed changes over time likely reflect a combination of true variation and substantial methodological changes across studies, instead of true changes in disease burden. Changes in diagnostic thresholds, improved detection and the number and breadth of conditions included in multimorbidity definitions likely contribute to the observed increase [6, 7, 48].

This review has several strengths, including a comprehensive and rigorous search strategy across four major databases and the absence of language restrictions, which maximized the identification of all relevant studies. The studies included in the review were all of moderate or high methodological quality. However, important limitations remain. The decision to restrict inclusion to studies published after 2015 was due to changes in diagnostic coding practices, the evolving epidemiology of pregnant women and recent conceptualization and operationalization of multimorbidity in pregnancy. However, this approach may have excluded earlier prevalence estimates and limits the historical coverage of this systematic review. Notably, substantial geographical gaps in the evidence base prevented us from estimating a truly global pooled prevalence of multimorbidity during pregnancy. No included studies originated from lower-middle income countries and LICs, and none were conducted in Africa or South America, limiting the representativeness and generalizability of current estimates. Pregnant women are often excluded from clinical studies, and much of the existing research focuses on pregnancy-specific conditions such as gestational diabetes, (pre-)eclampsia or peripartum mental health disorders [5]. While this body of work is essential, it tends to address conditions in isolation, and studies of the prevalence of multimorbidity during pregnancy are less common [5]. As a result, an important and complementary issue, multimorbidity during pregnancy, remains relatively underexplored in literature, especially in LMICs, despite its growing relevance in the context of changing maternal demographics and disease patterns. Furthermore, many excluded studies did not report multimorbidity in a format suitable for estimating prevalence, such as relying solely on comorbidity index scores, which substantially limited the number of eligible studies and resulted in inclusion of studies only from HICs. Moreover, the minimum sample size criterion of 200 participants, while intended to ensure estimate stability, may have excluded smaller studies from LMICs, potentially contributing to geographical imbalance in the pooled prevalence estimates. Notably, only one study was excluded during full-text screening on the basis of insufficient sample size, suggesting the practical impact of this criterion was limited. Another critical limitation is the absence of a standardized definition of multimorbidity in pregnancy, with studies using widely varying conditions lists and thresholds, which contributed to substantial variability in reported prevalences. These results strongly reinforce the need for consensus on a standardized operational definition of multimorbidity during pregnancy, without which meaningful comparisons across studies, populations, and regions remain fundamentally limited. This review also highlighted the need to increase reporting of multimorbidity among pregnant women, especially in low- and middle-income settings.

## Conclusion

This systematic review and meta-analysis indicated that multimorbidity during pregnancy is a significant but under-researched global health concern. Although the pooled prevalence across included studies was 7.18%, this estimate should be interpreted with caution due to substantial heterogeneity across studies, including differences in multimorbidity definitions, condition groupings, ascertainment methods, and study populations. The current evidence base is heavily weighted toward HICs and remains limited in low- and middle-income settings, restricting the ability to derive a fully representative global estimate. Despite these limitations, the findings underscore the burden of multimorbidity during pregnancy and the need for standardized definitions, improved measurement, and more geographically diverse research to inform coordinated, multidisciplinary models of antenatal care.

## Supplementary Information

Supplementary Material 1.

## Data Availability

Because this meta-analysis was based on data extracted from previously published research, most of the data and study materials are available in the public domain. The complete extracted data and code are available from the corresponding author upon reasonable request.

## Abbreviations

NCD: Non-communicable disease
HIC: High-income country
LMIC: Low- and middle-income country
LIC: Low-income country
UMIC: Upper-middle income country
PRISMA: Preferred Reporting Items for Systematic Review and Meta-Analysis
JBI: Joanna Briggs Institute
