# Supplementary material for: Global prevalence of multimorbidity during pregnancy: a systematic review and meta-analysis
Source: Reprod Health. 2026 Jul 27;23:147. doi: 10.1186/s12978-026-02424-8 (PMC13404628; doi:10.1186/s12978-026-02424-8)

### Supplementary materials

Supplement 1 Full search terms for Ovid Medline

| ***Medline*** | | | |
| --- | --- | --- | --- |
| **Interface**: Ovid Medline(R) ALL content coverage from January 1, 2015  **Date of search**: 26 September 2025  **Number of hits**: 2,391 | | **Field labels:**   - exp/ = exploded MeSH term - .ti,ab,kw = title, abstract and author keywords - * = truncation of word for alternate endings - # = 1 letter/number | |
| *#1* | exp Pregnancy/ or exp Pregnant Women/ or exp Maternal Health/ or (pregnancy or pregnan* or maternal or antenatal or antepartum or prenatal or gestation* or trimester or perinatal or birth or gravid* or obstetric*).ti,ab,kw | | 1,650,929 |
| *#2* | exp Multimorbidity/ or exp Comorbidity/ or (multimorbid* or multi-morbid* or comorbid* or co-morbid* or "multiple chronic condition*" or "multiple condition*").ti,ab,kw | | 402,459 |
| *#3* | exp Prevalence/ or exp Proportion/ or (prevalence or proportion*).ti,ab,kw | | 1,816,519 |
| *#4* | 1 and 2 and 3 | | 3,667 |
| *#5* | limit 4 to yr=”2015-Current” | | 2,391 |

Supplement 2 PRISMA 2020 Checklist

| **Section and topic** | **Item** | **Checklist item** | **Page** |
| --- | --- | --- | --- |
| ***TITLE*** | | | |
| Title | 1 | Identify the report as a systematic review | 1 |
| ***ABSTRACT*** | | | |
| Abstract | 2 | See the PRISMA 2020 for Abstracts checklist | 3-4 |
| ***INTRODUCTION*** | | | |
| Rationale | 3 | Describe the rationale for the review in the context of existing knowledge | 5-7 |
| Objectives | 4 | Provide an explicit statement of the objective(s) or question(s) the review addresses | 7 |
| ***METHODS*** | | | |
| Eligibility criteria | 5 | Specify the inclusion and exclusion criteria for the review and how studies were grouped for the syntheses. | 8 |
| Information sources | 6 | Specify all databases, registers, websites, organisations, reference lists and other sources searched or consulted to identify studies. Specify the date when each source was last searched or consulted. | 8 |
| Search strategy | 7 | Present the full search strategies for all databases, registers and websites, including any filters and limits used. | 8 |
| Selection process | 8 | Specify the methods used to decide whether a study met the inclusion criteria of the review, including how many reviewers screened each record and each report retrieved, whether they worked independently, and if applicable, details of automation tools used in the process. | 8-9 |
| Data collection process | 9 | Specify the methods used to collect data from reports, including how many reviewers collected data from each report, whether they worked independently, any processes for obtaining or confirming data from study investigators, and if applicable, details of automation tools used in the process. | 9-10 |
| Data items | 10a | List and define all outcomes for which data were sought. Specify whether all results that were compatible with each outcome domain in each study were sought (e.g. for all measures, time points, analyses), and if not, the methods used to decide which results to collect. | 9-10 |
|  | 10b | List and define all other variables for which data were sought (e.g. participant and intervention characteristics, funding sources). Describe any assumptions made about any missing or unclear information. | 10 |
| Study risk of bias | 11 | Specify the methods used to assess risk of bias in the included studies, including details of the tool(s) used, how many reviewers assessed each study and whether they worked independently, and if applicable, details of automation tools used in the process. | 9-10 |
| Effect measures | 12 | Specify for each outcome the effect measure(s) (e.g. risk ratio, mean difference) used in the synthesis or presentation of results. | 9-10 |
| Synthesis methods | 13a | Describe the processes used to decide which studies were eligible for each synthesis (e.g. tabulating the study intervention characteristics and comparing against the planned groups for each synthesis (item #5)). | 9-10 |
|  | 13b | Describe any methods required to prepare the data for presentation or synthesis, such as handling of missing summary statistics, or data conversions. | 9-10 |
|  | 13c | Describe any methods used to tabulate or visually display results of individual studies and syntheses. | 9-10 |
|  | 13d | Describe any methods used to synthesize results and provide a rationale for the choice(s). If meta-analysis was performed, describe the model(s), method(s) to identify the presence and extent of statistical heterogeneity, and software package(s) used. | 9-10 |
|  | 13e | Describe any methods used to explore possible causes of heterogeneity among study results (e.g. subgroup analysis, meta-regression). | 9-10 |
|  | 13f | Describe any sensitivity analyses conducted to assess robustness of the synthesized results. | 9-10 |
| Reporting bias assessment | 14 | Describe any methods used to assess risk of bias due to missing results in a synthesis (arising from reporting biases). | 9-10 |
| Certainty assessment | 15 | Describe any methods used to assess certainty (or confidence) in the body of evidence for an outcome. | 9-10 |
| ***RESULTS*** | | | |
| Study selection | 16a | Describe the results of the search and selection process, from the number of records identified in the search to the number of studies included in the review, ideally using a flow diagram. | 11-12 |
|  | 16b | Cite studies that might appear to meet the inclusion criteria, but which were excluded, and explain why they were excluded. | 11 |
| Study characteristics | 17 | Cite each included study and present its characteristics. | 14-16 |
| Risk of bias in studies | 18 | Present assessments of risk of bias for each included study. | Supplement 6 |
| Results of individual studies | 19 | For all outcomes, present, for each study: (a) summary statistics for each group (where appropriate) and (b) an effect estimate and its precision (e.g. confidence/credible interval), ideally using structured tables or plots. | 11-20 |
| Results of syntheses | 20a | For each synthesis, briefly summarise the characteristics and risk of bias among contributing studies. | 11-20 |
|  | 20b | Present results of all statistical syntheses conducted. If meta-analysis was done, present for each the summary estimate and its precision (e.g. confidence/credible interval) and measures of statistical heterogeneity. If comparing groups, describe the direction of the effect. | 13, 17-20 |
|  | 20c | Present results of all investigations of possible causes of heterogeneity among study results. | 18-20 |
|  | 20d | Present results of all sensitivity analyses conducted to assess the robustness of the synthesized results. | 19-20 |
| Reporting biases | 21 | Present assessments of risk of bias due to missing results (arising from reporting biases) for each synthesis assessed. | N/A |
| Certainty of evidence | 22 | Present assessments of certainty (or confidence) in the body of evidence for each outcome assessed. | N/A |
| ***DISCUSSION*** | | | |
| Discussion | 23a | Provide a general interpretation of the results in the context of other evidence. | 21-24 |
|  | 23b | Discuss any limitations of the evidence included in the review. | 23-24 |
|  | 23c | Discuss any limitations of the review processes used. | 23-24 |
|  | 23d | Discuss implications of the results for practice, policy, and future research. | 24 |
| ***OTHER INFORMATION*** | | | |
| Registration and protocol | 24a | Provide registration information for the review, including register name and registration number, or state that the review was not registered. | 9 |
|  | 24b | Indicate where the review protocol can be accessed, or state that a protocol was not prepared. | 9 |
|  | 24c | Describe and explain any amendments to information provided at registration or in the protocol. | 9 |
| Support | 25 | Describe sources of financial or non-financial support for the review, and the role of the funders or sponsors in the review. | 26 |
| Competing interests | 26 | Declare any competing interests of review authors. | 26 |
| Availability of data, code and other materials | 27 | Report which of the following are publicly available and where they can be found: template data collection forms; data extracted from included studies; data used for all analyses; analytic code; any other materials used in the review. | 26 |

Supplement 3 Data extraction tool

| **Data extraction item** | **Additional information** |
| --- | --- |
| ***General information and context*** | |
| *Title* | Free text |
| *Lead author name and year of publication* | e.g. Smith 2018 |
| *Country* | e.g. Kenya |
| *Geographic region* | North America, South America, Africa, Europe, Asia, Oceania, Antarctica |
| *World Bank income group* | Low-, lower-middle, upper-middle or high-income |
| ***Methods*** | |
| *Study design* | Cohort study, cross-sectional study or other |
| *Start date – end date* | e.g. 2020-2023 |
| *Data source* | e.g. National Health Survey database |
| *Method of recruitment of participants* | Phone, mail, clinic patients, voluntary, routinely collected data or other |
| ***Participants*** | |
| *Population description* | Information about pregnancy, e.g. singleton |
| *Total number of participants* | Total number of pregnant women |
| *Mean age in years* | If missing, input “999” |
| *Median age in years* | If missing, input “999” |
| *Women in age group category ≥ 35 years* | As n (%), e.g. 100 (25%) |
| ***Multimorbidity*** | |
| *Definition of multimorbidity* | e.g. two or more diseases |
| *Number of conditions included in multimorbidity definition* | e.g. 2 |
| *Number of conditions in the definition list of multimorbidity* | e.g. 79; if missing, input “999” |
| *Conditions in multimorbidity definition* | e.g. depression, cancer |
| *Definitions used for conditions* | e.g. EDS score ≥ 10 |
| *Mental health conditions reported as part of multimorbidity* | Yes/no |
| *Ascertainment of exposure* | Objective, self-reported |
| *Context of exposure* | e.g. pre-pregnancy, pregnancy-related, post-partum |
| *Point in time when multimorbidity was ascertained* | e.g. in 5 years prior to conception, or at the point of conception |
| *Pregnancy trimester* | Pre-pregnancy, first, second, third, post-partum, not specified |
| ***Outcome*** | |
| *Number of pregnant women with the most common multimorbidity* | Numerator |
| *Prevalence of multimorbidity* | As n (%), e.g. 100 (25%) |
| *Groups in which multimorbidity is more common* | Were there groups in which multimorbidity was more common? e.g. based on SES, ethnicity or age |
| *Additional comments* | Free text |

Supplement 4 Quality assessment tool

Based on JBI Critical Appraisal Tool for Prevalence Studies ^20^

| **Participant selection** | | | |
| --- | --- | --- | --- |
| ***Was the sample frame appropriate to address the target population?*** | | | |
| Yes | No | Unclear | Not applicable |
| ***Were study participants sampled in an appropriate way?*** | | | |
| Yes | No | Unclear | Not applicable |
| ***Was the sample size adequate?*** | | | |
| Yes | No | Unclear | Not applicable |
| **Methods** | | | |
| ***Were the study subjects and the setting described in detail?*** | | | |
| Yes | No | Unclear | Not applicable |
| ***Was the data analysis conducted with sufficient coverage of the identified sample?*** | | | |
| Yes | No | Unclear | Not applicable |
| ***Was there appropriate statistical analysis?*** | | | |
| Yes | No | Unclear | Not applicable |
| ***Was the response rate adequate, and if not, was the low response rate managed appropriately?*** | | | |
| Yes | No | Unclear | Not applicable |
| **Measurement of outcome** | | | |
| ***Was the condition measured in a standard, reliable way for all participants?*** | | | |
| Yes | No | Unclear | Not applicable |
| ***Were valid methods used for the identification of the condition?*** | | | |
| Yes | No | Unclear | Not applicable |

Supplement 5 Characteristics of included studies (continuation of Table 1)

| **Author and year (ref)** | **Population description** | **Mean/median age (years)** | **No of conditions in definition** | **Women** $\geq$ **35 years old (n (%))** | **Conditions in definition list of multimorbidity** | **Narrative definition of multimorbidity** | **Type of conditions** |
| --- | --- | --- | --- | --- | --- | --- | --- |
| Akagi 2024 ^15^ | Fetal records of women’s first live birth within the study period, who had a singleton pregnancy without chromosomal abnormalities | N/A | 2 | 22,786 (27.5%) | Allergic diseases, such as asthma, anemia, diabetes mellitus, dyslipidaemia, epilepsy, gastric or duodenal ulcers, heart disease, hepatitis, HIV infection, hypertension, inflammatory bowel disease, kidney disease, malignancy, migraine, neurologic disease, other sexually transmitted diseases (Chlamydia trachomatis and syphilis), mental disorders, rheumatic or collagen diseases and thyroid disease, episode of domestic violence, substance abuse, being obese (BMI≥25), and being thin (BMI<18.5). | "Multimorbidity was defined as the coexistence of two or more physical, mental or social conditions in an individual according to previous reports. To identify pregnant women with the disease more rigorously, the diseases of pregnant women were defined as those that were medically treated at the time of pregnancy." | Chronic mental and physical conditions |
| Akaishi 2023 ^21^ | Hospitalization episodes with child delivery and women who were evaluated for psychiatric outcomes | Median: 33 | 2 | N/A | Depression, schizophrenia, alcohol use disorder, personality disorder, insomnia, anxiety disorders, bipolar disorder, adjustment disorder, previous suicide attempt | “Two or more prenatal psychiatric problems” | Chronic mental conditions |
| Aubry 2019 ^22^ | Women in Switzerland who delivered singleton infants between 22 and 43 weeks gestation from January 1, 2005 to December 31, 2016 | Mean: 31.06 | 2 | N/A | Pre-existing diabetes, gestational diabetes, gestational hypertension without significant proteinuria (≥140/90), preeclampsia, eclampsia, pre-existing hypertension | “> 1 comorbidities” | Chronic physical and pregnancy-specific conditions |
| Aumais 2025 ^23^ | English- or French speaking women over 18 who were either pregnant or within six months postpartum and have met with a perinatal mental health professional at a participating site between July 2018 and July 2024. | Mean: 32.7 | 2 | N/A | Anxiety disorders, depressive disorders, adjustment disorder,  trauma and stressor-related disorders,  personality disorders, obsessive-compulsive and related disorders,  attention-deficit/ hyperactivity disorder, bipolar and related disorders,  substance use disorder, eating disorders, schizophrenia spectrum and other psychotic disorders, somatic disorders | “Participants had more than one concomitant diagnosis" | Chronic mental conditions |
| Azcoaga-Lorenzo 2023 ^14^ | Singleton pregnancies $\geq$24 weeks at recruitment, among women aged 15-49. | N/A | 2 | 4,656 (16.8%) | Pre-pregnancy conditions in MuM-PreDiCT consortium | "Multimorbidity was defined by the presence of two or more pre-existing long-term physical or mental health conditions. Multimorbidity was calculated at the estimated time of conception for each pregnancy using a predefined list of 79 conditions published by the MuM-PreDiCT consortium." | Chronic mental and physical conditions |
| Belsti 2025 ^24^ | Pregnant women who received care at Monash Health maternity hospitals in Melbourne, Australia, between January 2016 and June 2021. | N/A | 2 | 10,913 (22.5%) | Vitamin deficiency, overweight, obesity, mental disorders, other blood disorders, asthma, thyroid disease, CVD, urinal disease, Diabetes Mellitus, PCOS, medical surgical, iron deficiency anemia, migraine, UTI, musculoskeletal disease, kidney disease, bowel disease, autoimmune disease, vitamin deficiency, liver disease, pregnancy related complications, respiratory disease, herpes, genital disease | “Two or more medical conditions.” | Chronic physical and pregnancy-specific conditions |
| Brown 2024 ^8^ | Women aged 15-55 years old, with a singleton or twin pregnancy. | Mean: 29.2 | 2 | 69,024 (21.3%) | Asthma, cancer, cardiac arrhythmia, chronic hypertension, chronic liver disease, COPD, congestive heart failure, coronary syndrome, diabetes mellitus, inflammatory bowel disease, mood and anxiety disorders, osteoarthritis, other mental illness, psychotic mental illness, renal failure, stroke and substance-use disorders, HIV, MS, obesity, RA, SLE | "Multiple chronic conditions (MCC), defined as having two or more chronic conditions, as ascertained in the 5-year period before the estimated date of conception for the index pregnancy. Consistent with the Quality and Outcomes Framework, we adopted the 17 chronic conditions used in prior MCC research.” | Chronic mental and physical conditions |
| Brown 2025 ^16^ | Females aged 13-54 years, who had a recognized pregnancy between April 1, 2012 and March 31, 2021, and, as residents of Ontario, were eligible for Ontario health insurance in the 2 years before the estimated date of conception | Mean: 29.34-30.34 | 2 | 307,098 (22.4%) | Alcohol and substance use disorders, asthma, cancer, cardiac arrhythmia, chronic hypertension, chronic kidney disease, chronic liver disease, COPD, congestive heart failure, coronary artery syndrome, DM, HIV, inflammatory bowel disease, migraine, mood and anxiety disorders, multiple sclerosis, obesity, osteoarthritis, other mental illness, psychotic disorders, rheumatoid arthritis, stroke and SLE | “MCC subdivided into non-complex and complex MCC. Non-complex = 2 chronic conditions Complex MCC was defined as having ≥3 chronic conditions affecting ≥3 body systems, with body systems defined by the chapters of the International Classification of Diseases and Related Health Problems, version 10.” | Chronic mental and physical conditions |
| Chhabria 2024 ^25^ | Women, aged 14-54 years, who a hospital admission for labor and delivery between January 1st, 2014 - December 31st, 2019, and who had continuous enrollment one year prior to and 1-year postpartum, with no more than 90 days of an enrollment gap. | Mean: 31.41-32.32 | 2 | N/A | Mental illness: Depression, anxiety, bipolar, psychosis  MetS-C: obesity, diabetes, hypertension, low HDL, triglycerides | “1 or more metabolic syndrome conditions (MetS-C) + 1 or more maternal mental illness OR Two or more MetS-C, prior to delivery” | Chronic mental and physical conditions |
| Dikmen-Yildiz 2017 ^26^ | Turkish-speaking pregnant women aged 18 or over recruited from three hospitals, who had routine antenatal appointments in gestational week 26-35. Only women with a live birth were included. | Mean: 27.6 | 3 | 107 (11.3%) | Depression, anxiety and PTSD | “Co-existence of depression, anxiety and PTSD” | Chronic mental conditions |
| Hohmann-Marriott 2019 ^27^ | Pregnant women due to give birth in 2008/2009 | N/A | 2 | N/A | Diabetes, heart disease, high blood pressure, asthma, depression, anxiety/panic attacks | “Multimorbidity (more than one condition)” | Chronic mental and physical conditions |
| Kent 2025 ^6^ | Pregnant women aged 15-49 at birth, with a start date of pregnancy between 1st January 2012 and 31st December 2021, with a valid HCN recorded in NIMATS. | N/A | 2 | 31,795 (23.08%) | Long-term physical and mental conditions, using MuM-PreDiCT phenome | "Presence of two or more physical or mental health conditions, and further stratified by physical health multimorbidity, mental health multimorbidity, and multimorbidity including both physical and mental health conditions" | Chronic mental and physical conditions |
| Lau 2025 ^28^ | Pregnant women aged 18-59 years with pregnancies with gestational age of at least 21 weeks, who had a live birth | Mean: 33 | 4 | N/A | Myocardial infarction, heart failure, vascular dissection, thromboembolism (venous, pulmonary or systematic), cerebrovascular disease (TIA or hemorrhagic or ischemic cerebrovascular attack), ventricular or atrial arrhythmias, cardiac arrest, chronic hypertension, gestational hypertension, superimposed preeclampsia, eclampsia and HELLP syndrome | “More than 3 cardiovascular comorbidities” | Chronic physical and pregnancy-specific conditions |
| Lee 2022 ^29^ | First pregnancy of women aged 15-49 years with a conception data in 2018. | N/A | 2 | 7,576 (20.1%) | Long-term health conditions determined from Read Codes version 2 and ICD-10 | "Multimorbidity was defined by the presence of two or more pre-existing long-term physical or mental health conditions prior to the index pregnancy. We defined long-term conditions as conditions that have ongoing significant impact on patients, including conditions that are relapsing and remitting in nature. One of the wider research aims is to mitigate the effect of multimorbidity on adverse pregnancy outcomes. As pregnancy related conditions (e.g., gestational diabetes and pregnancy induced hypertension) will be subsequently studied as maternal outcomes, they were not included in the definition of pre-existing multimorbidity." | Chronic mental and physical conditions |
| Nakanishi 2023 ^30^ | Fetal records of women's first singleton live birth within the study period. Pregnancies with chromosomal abnormalities and missing values were excluded | Median: 31 | 2 | 23,721 (27.3%) | Allergic diseases such as asthma, anemia, diabetes mellitus, dyslipidaemia, epilepsy, gastric or duodenal ulcer, heart disease, hepatitis, HIV infection, hypertension, inflammatory bowel disease, kidney disease, malignancy, migraine, neurological disease, other sexually transmitted diseases (Chlamydia trachomatis and syphilis), psychiatric disorders, rheumatic or collagen diseases, and thyroid disease, abnormal pre-pregnancy BMI (including underweight and obesity), physical or verbal domestic violence from intimate partners, and substance abuse. | "Coexistence of two or more physical, psychological or social conditions in an individual. A maternal chronic condition was defined as a condition that was medically treated at the time of pregnancy" | Chronic mental and physical conditions |
| Stanhope 2022 ^31^ | Singleton births within the study period between October 2014 and July 2021. | Mean: 27.4 | 2 | 2,178 (15.4%) | Psychiatric history, preexisting cardiac disease, HIV, connective tissue or autoimmune disease, bleeding disorder, chronic kidney disease, pulmonary hypertension, chronic diabetes (type I or II), chronic hypertension, seizure disorders and asthma | “Classifying patients with two or more different diagnoses as having multimorbidity. The main definition included all the comorbidities described.” | Chronic mental and physical conditions |
| Subramanian 2023 ^32^ | Women aged 15-49 with a pregnancy episode within the eligibility period, who were registered to an eligible general practice for a minimum of 12 months | N/A | 2 | 134,377 (16.5%) | Conditions in MuM-PreDiCT phenome | "For this study, multimorbidity was defined as having 2 or more long-term physical or mental health conditions at the start of pregnancy. For long-term conditions that are episodic in nature, such as eczema, we considered them to be actively present at the start of pregnancy if they were diagnosed or treated for the same in the previous 12 months.” | Chronic mental and physical conditions |
| Thompson 2022 ^33^ | Women over 18 years old with singleton or multiple birth within the 12 year study period (2001-2012), and were reported in the Inpatient Hospital claims within the MAX files. | Mean: 26-27 | 2 | 854,568 (11.1%) | 1) Congestive heart failure 2) Pulmonary disorder (any) 3) Peripheral vascular disease 4) Hypertension 5) Diabetes mellitus (all types) 6) Myocardial infarction/Coronary artery disease 7) Pancreatitis 8) Schizophrenia 9) Schizoaffective disorder 10) Other psychosis 11) Bipolar disorder 12) Major depression 13) Other depression 14) Post-traumatic stress disorder 15) Stroke (any) 16) Renal insufficiency (any) 17) Any hepatitis B 18) Any hepatitis C 19) Most anemias 20) Hyperlipidemia 21) Urine/kidney stone(s) 22) Cirrhosis of the liver 23) Decompensated liver disease 24) End-stage liver disease 25) Any drug abuse 26) Any alcohol abuse 27) Any cancer | "CCs were identified as previously described and the number of individual conditions was classified as 0, 1, 2, 3, 4, or 5+ total conditions. We examined total number of comorbid conditions per enrollee out of 27 total comorbid conditions. To identify these comorbidities, we used the classification system developed by the VACS. For each comorbid condition, we required 2 outpatient claims or 1 inpatient claim with evidence of the condition within the look-back period." | Chronic mental and physical conditions |

Supplement 6 Quality assessment details

Based on JBI Critical Appraisal Tool for Prevalence Studies ^20^

| **Author and year (ref)** | **Participant selection (3 points)** | **Methods (4 points)** | **Measurement of outcome (2 points)** | **Total points** | **Quality** |
| --- | --- | --- | --- | --- | --- |
| Akagi 2024 ^15^ | 6 | 8 | 4 | 18 | High |
| Akaishi 2023 ^21^ | 6 | 8 | 4 | 18 | High |
| Aubry 2019 ^22^ | 6 | 8 | 4 | 18 | High |
| Aumais 2025 ^23^ | 5 | 8 | 4 | 17 | High |
| Azcoaga-Lorenzo 2023 ^14^ | 6 | 8 | 4 | 18 | High |
| Belsti 2025 ^24^ | 6 | 6 | 4 | 16 | Moderate |
| Brown 2024 ^8^ | 6 | 8 | 4 | 18 | High |
| Brown 2025 ^16^ | 6 | 8 | 4 | 18 | High |
| Chhabria 2024 ^25^ | 6 | 8 | 4 | 18 | High |
| Dikmen-Yildiz 2017 ^26^ | 5 | 8 | 4 | 17 | High |
| Hohmann-Marriott 2019 ^27^ | 6 | 6 | 4 | 16 | Moderate |
| Kent 2025 ^6^ | 6 | 8 | 4 | 18 | High |
| Lau 2025 ^28^ | 6 | 8 | 4 | 18 | High |
| Lee 2022 ^29^ | 6 | 8 | 4 | 18 | High |
| Nakanishi 2023 ^30^ | 6 | 8 | 4 | 18 | High |
| Shuffrey 2022 ^36^ | 6 | 8 | 4 | 18 | High |
| Stanhope 2022 ^31^ | 6 | 8 | 4 | 18 | High |
| Subramanian 2023 ^32^ | 6 | 8 | 4 | 18 | High |
| Thompson 2022 ^33^ | 6 | 8 | 4 | 18 | High |

Supplement 7 Forest plots for subgroup analysis

**Supplement 7A Forest plot of multimorbidity prevalence by geographical region**

**
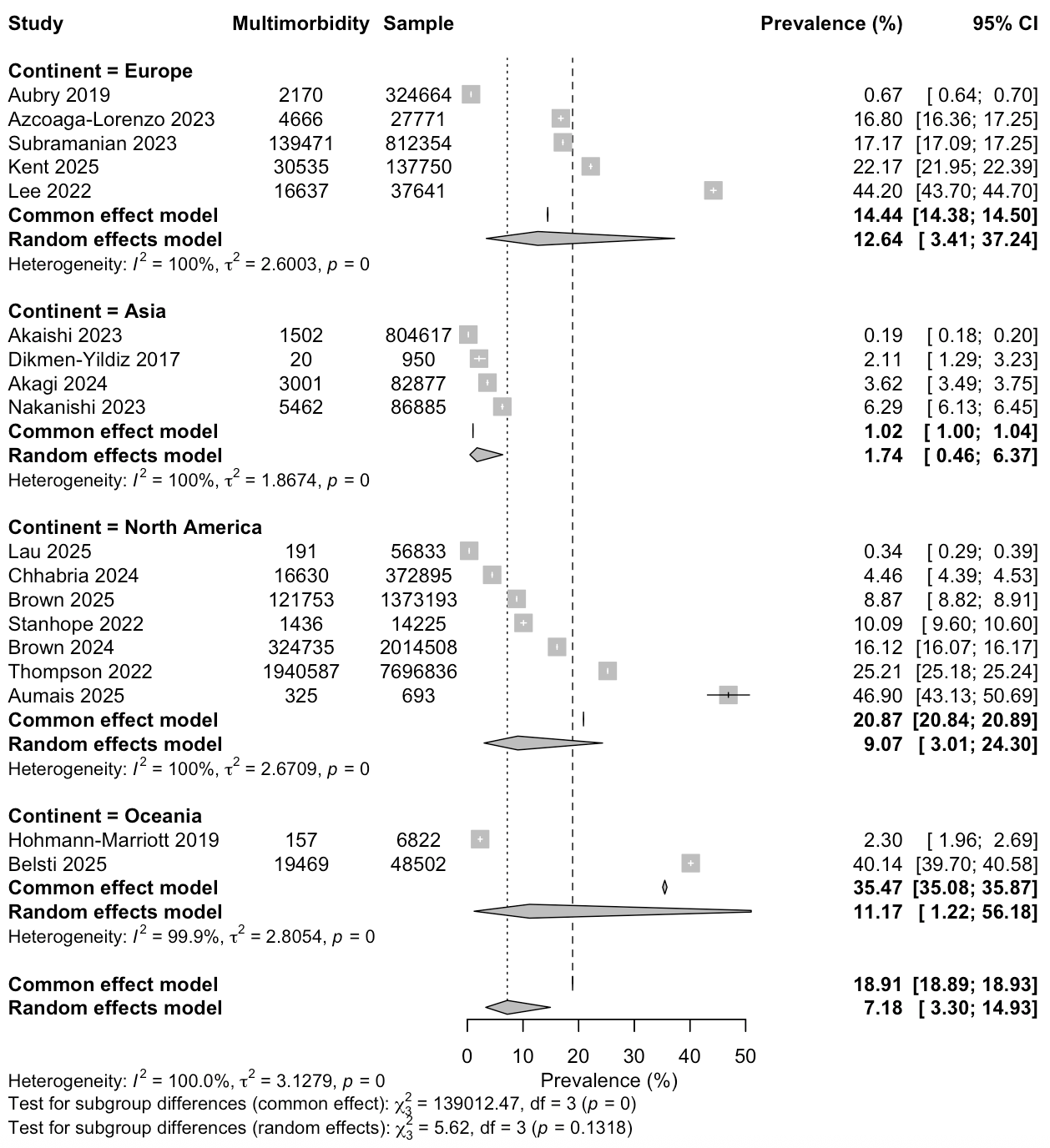
**

**Supplement 7B Forest plot of multimorbidity prevalence by study start year**

**
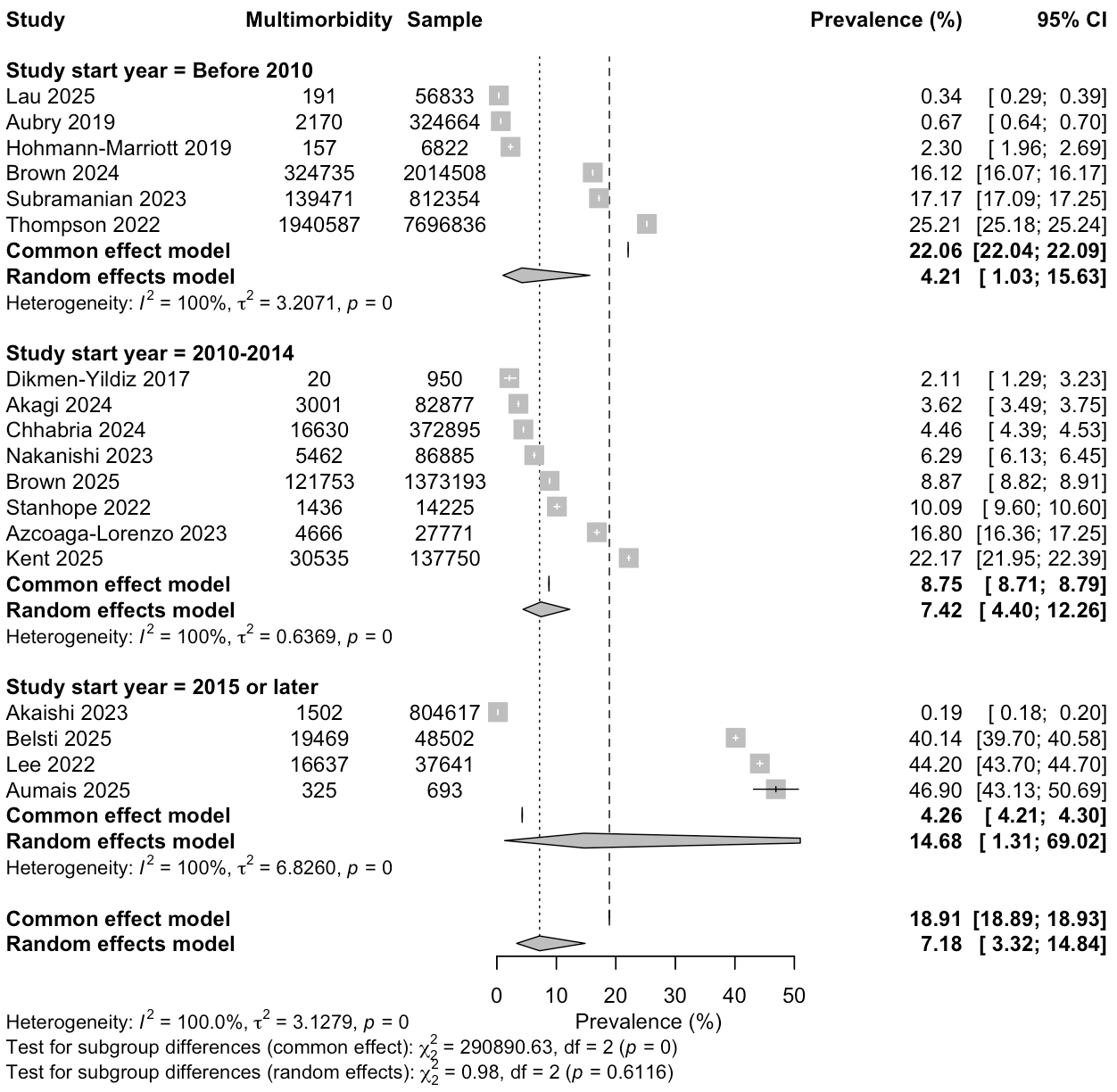
**

**Supplement 7C Forest plot of multimorbidity prevalence by study design**

**
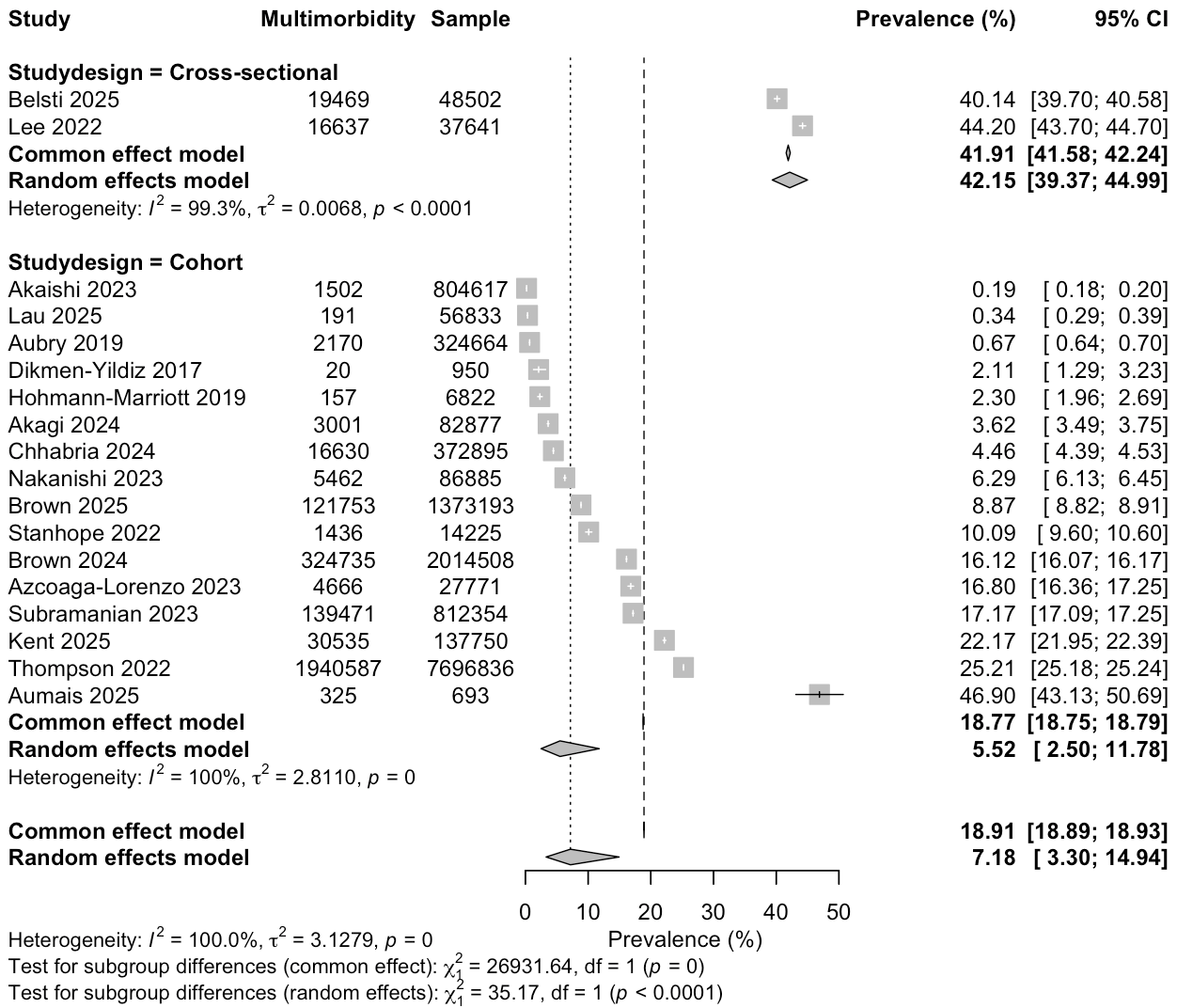
**

**Supplement 7D Forest plot of multimorbidity prevalence by sample size**

**
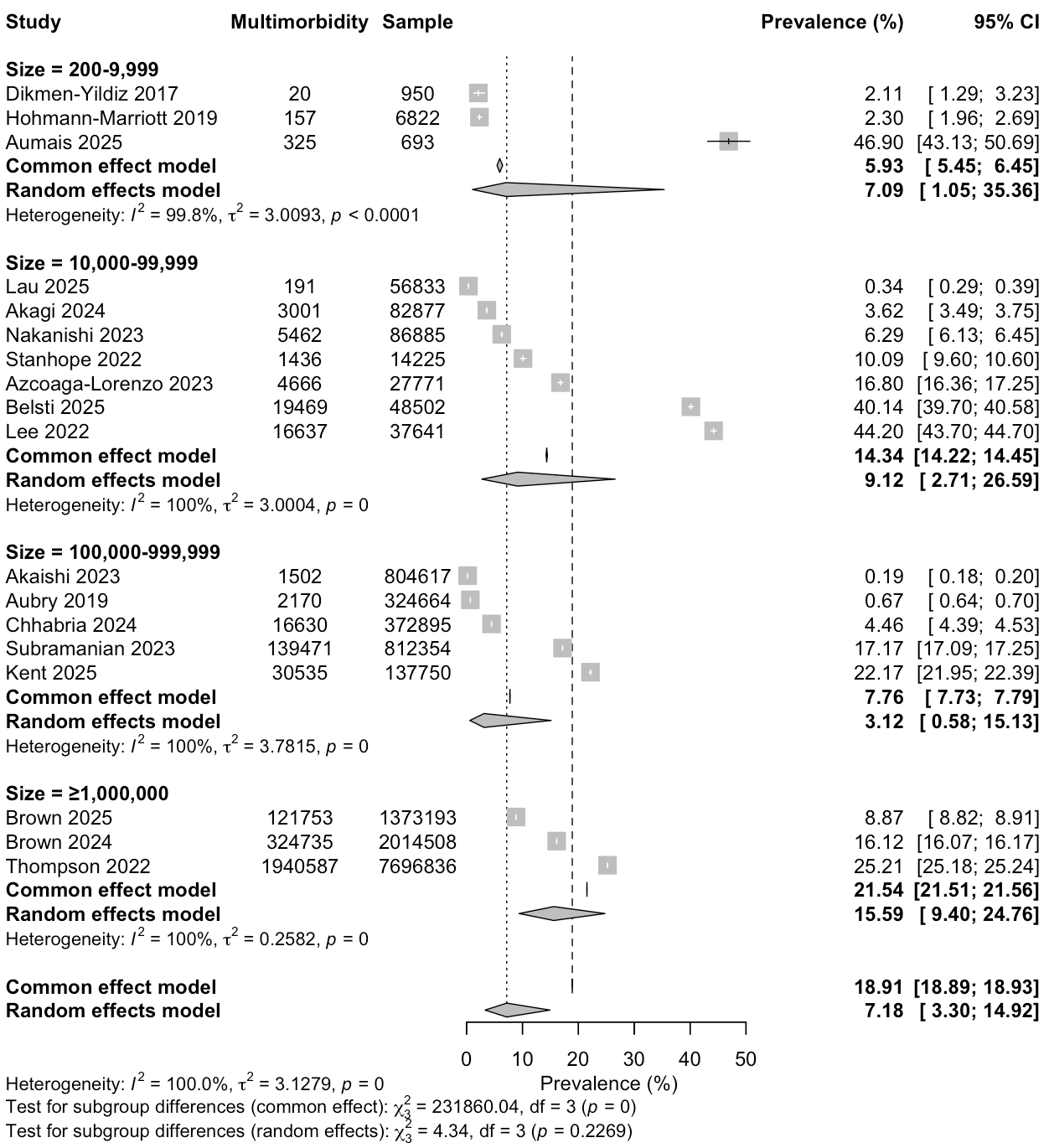
**

**Supplement 7E Forest plot of multimorbidity prevalence by number of conditions in multimorbidity definition**

**
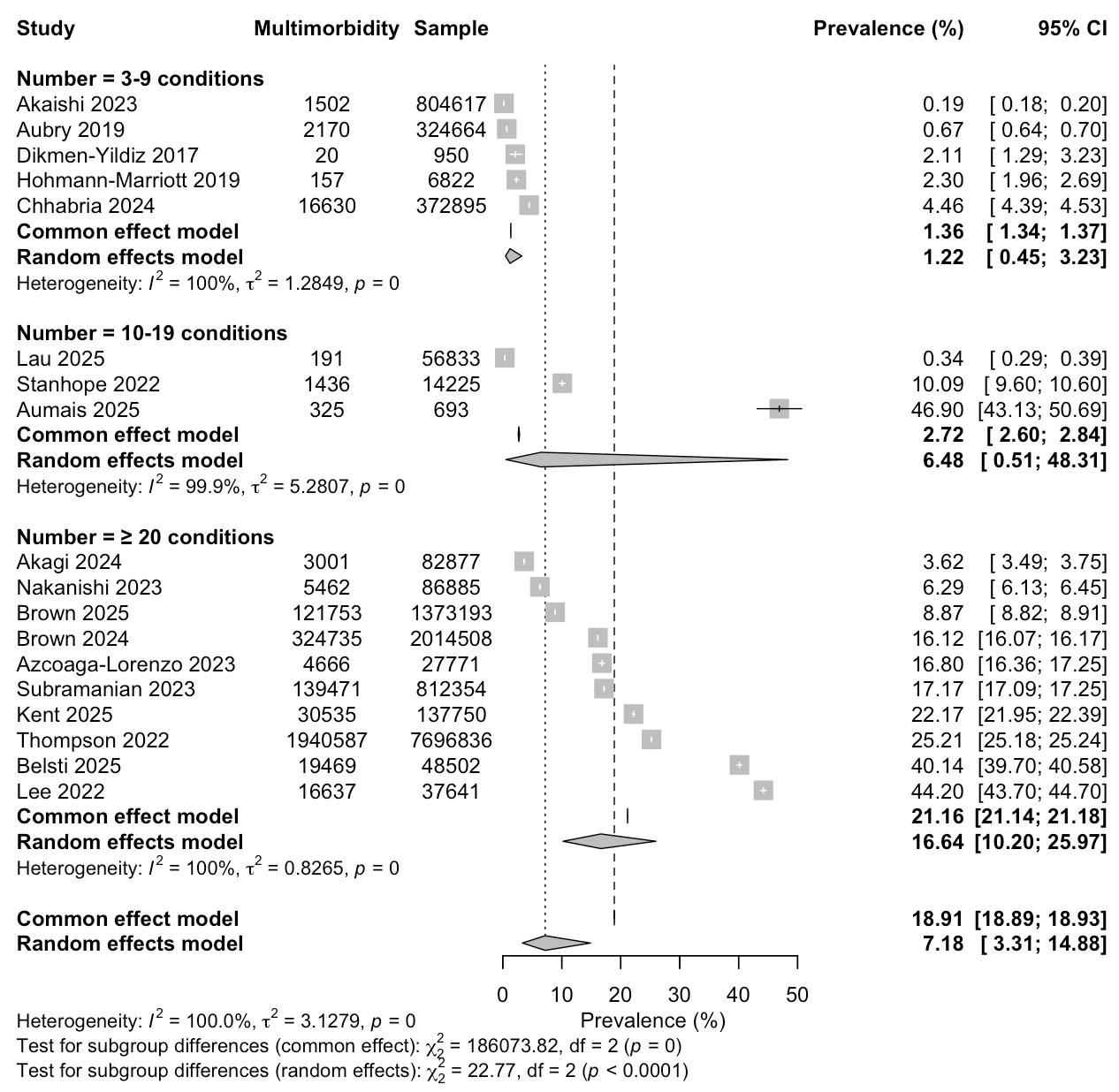
**

**Supplement 7F Forest plot of multimorbidity prevalence by types of conditions**

**
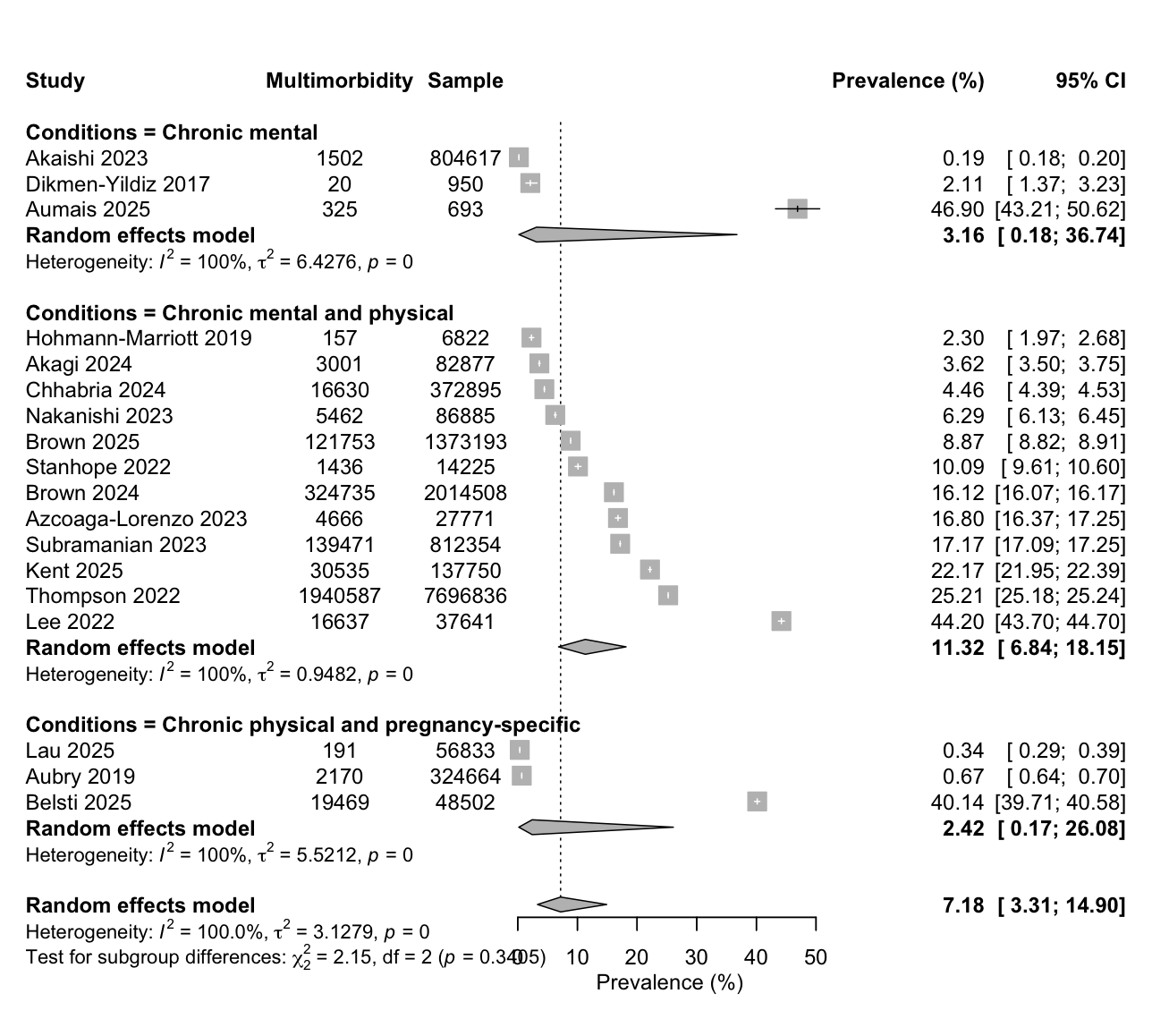
**

Supplement 8 Funnel plot and trim-and-fill analysis for publication bias


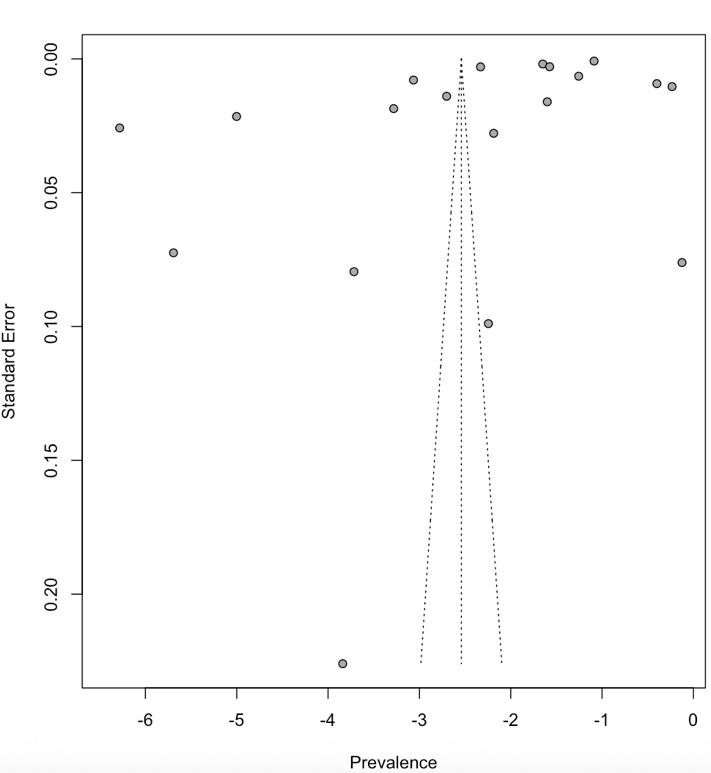

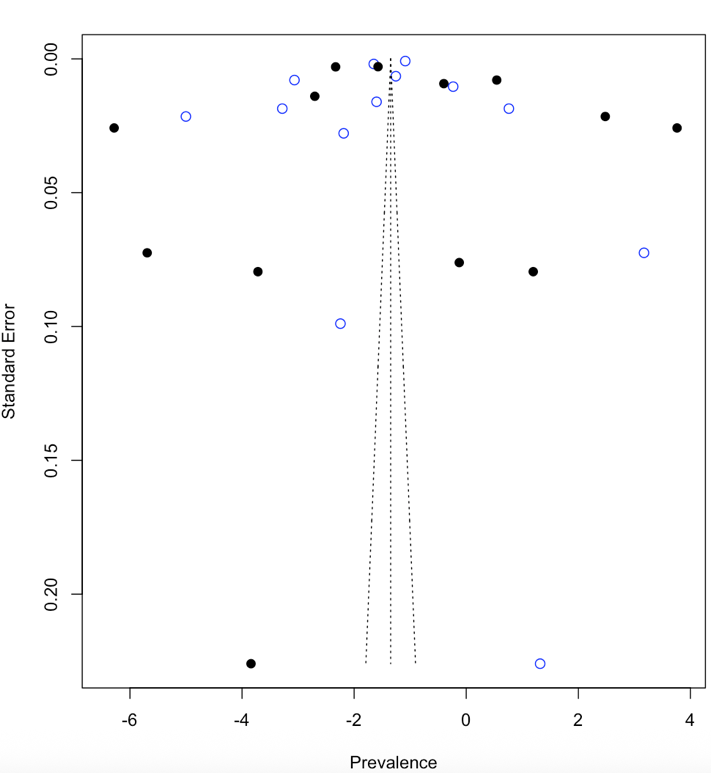


Supplement 9 Forest plots for sensitivity analysis

**Supplement 9A Forest plot excluding studies with < 10,000 participants (n = 15)**

**
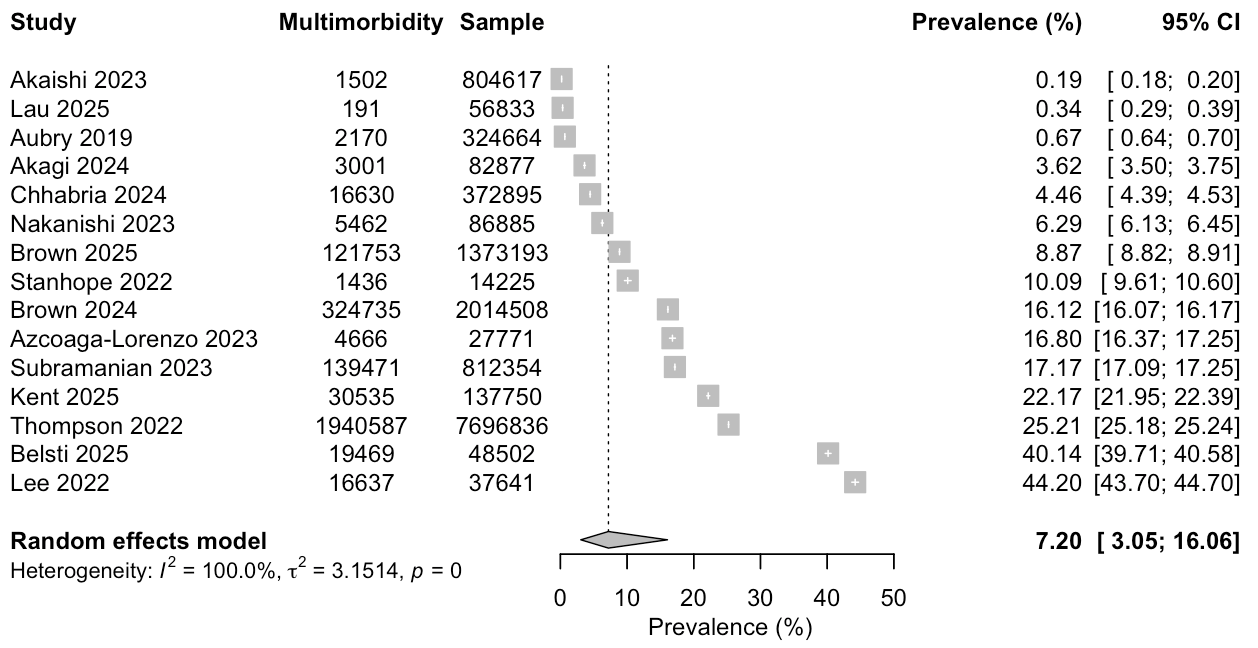
**

**Supplement 9B Forest plot with only cohort studies (n = 16)**

**
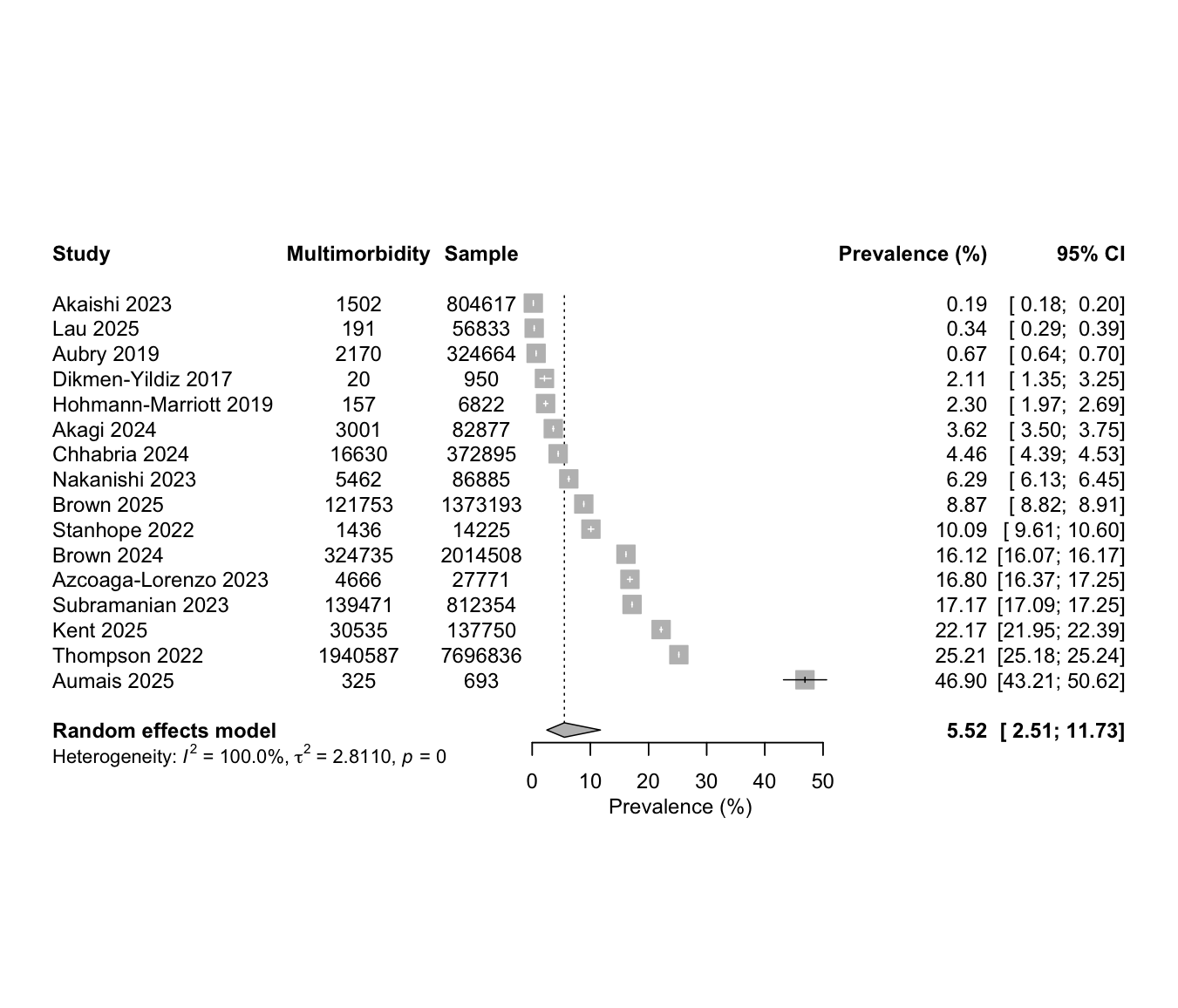
**

**Supplement 9C Forest plot excluding studies that defined multimorbidity using fewer than 10 conditions (n = 13)**

**
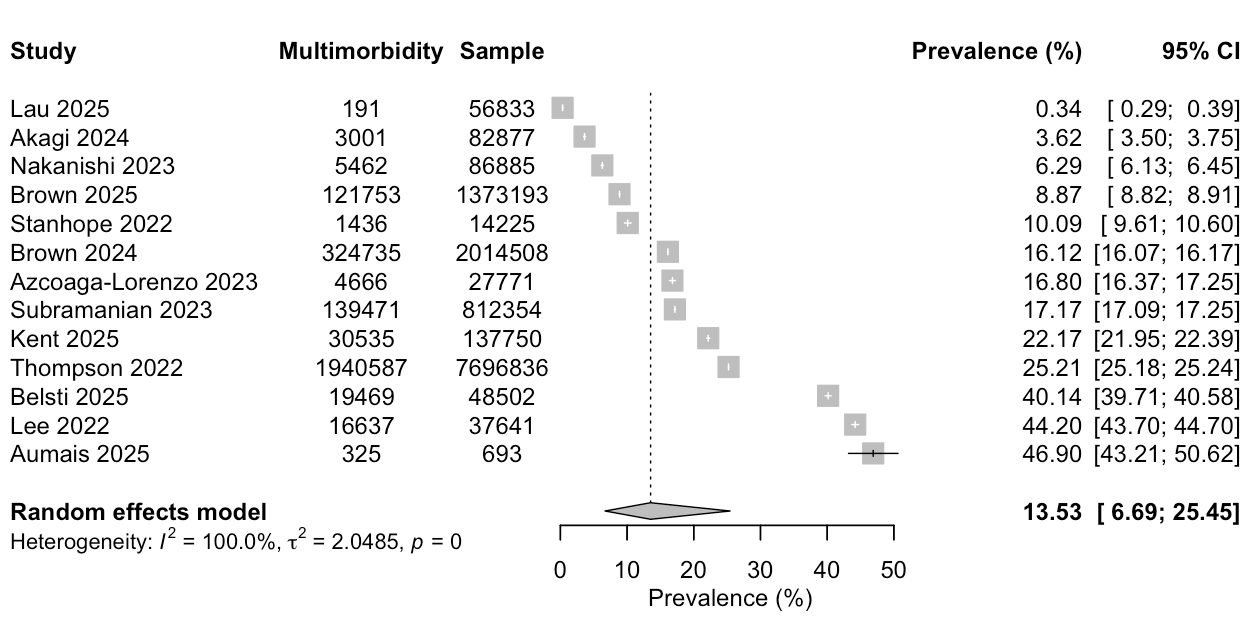
**

**Supplement 9D Forest plot of studies on both mental and physical conditions (n = 12)**

**
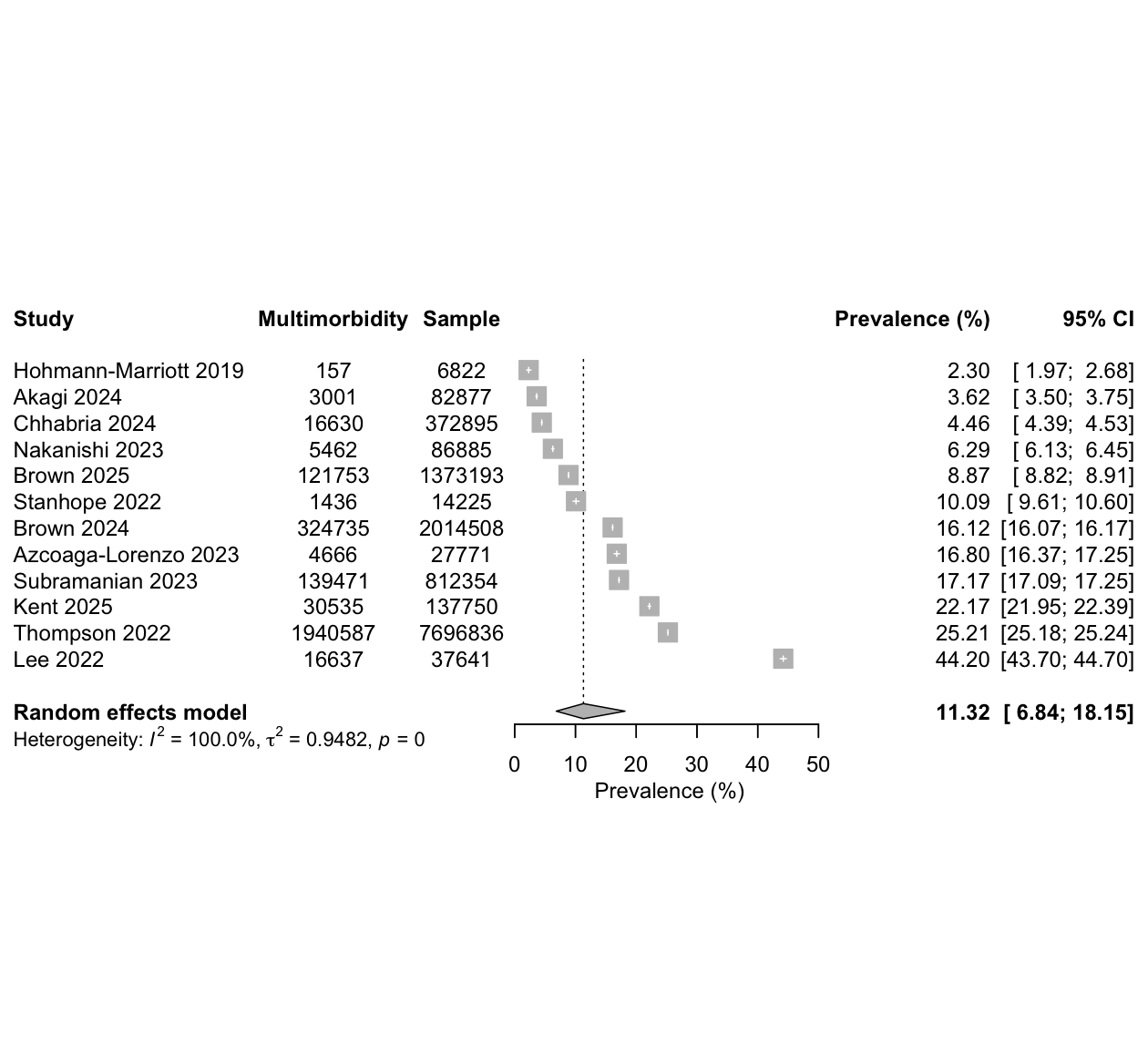
**

**Supplement 9E Forest plot of outlier analysis, excluding studies with extreme prevalence estimates outside the SD interval of 1.30% to 31.20% (n = 12)**

**
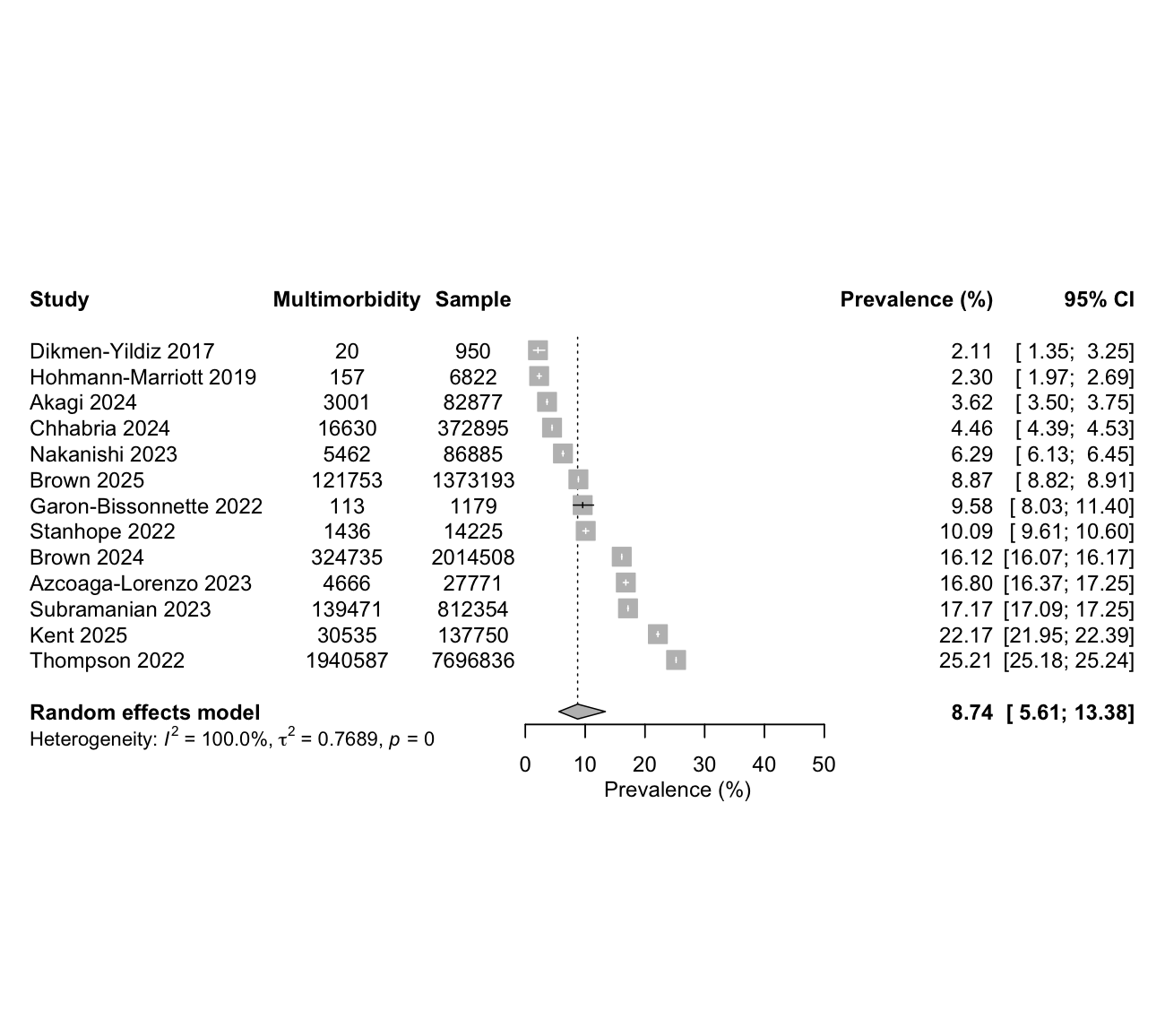
**

**Supplement 9F Forest plot of studies in high-income countries (n = 17)**


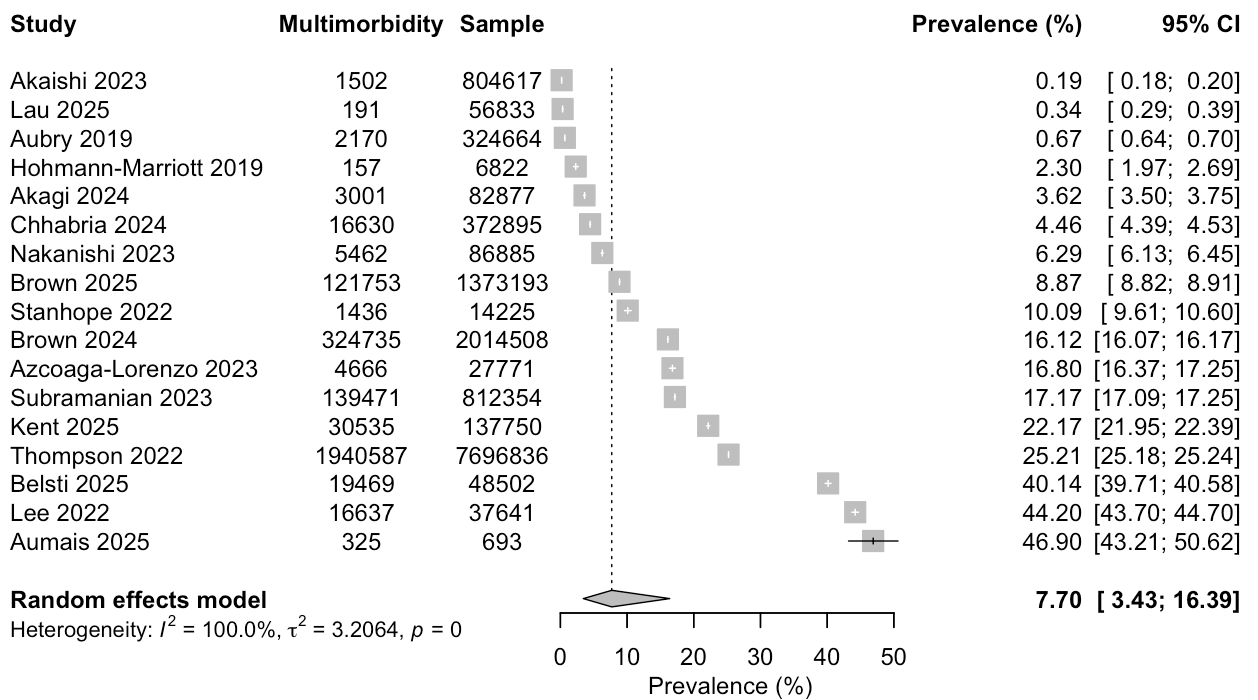

Supplement: Supplementary file 1 — Supplementary Material 1. [file 12978_2026_2424_MOESM1_ESM.docx]
